# Explainable Deep Tabular Learning for Credit Risk Assessment: An Information-Theoretic Cross-Attentional Transformer Approach

**DOI:** 10.3390/e28080837

**Published:** 2026-07-27

**Authors:** Bowen Dong, Xinyu Zhang, Ziwei Hong, Chaoya Yan, Weiyan Zhu, Lingmin Hou, Yifan Feng

**Affiliations:** 1 School of Electrical Automation and Information Engineering, Tianjin University, Tianjin 300072, China; 2 Department of Computer Science, Rochester Institute of Technology, Rochester, NY 14623, USA; 3 P.C. Rossin College of Engineering and Applied Science, Lehigh University, Bethlehem, PA 18015, USA; 4 Department of Computer Science, Rutgers University, New Brunswick, NJ 08901, USA; 5 Meta Platforms Inc., Menlo Park, CA 94025, USA; 6 Department of Computer Science and Engineering, Santa Clara University, Santa Clara, CA 95053, USA

**Keywords:** loan approval decision modeling, tabular transformer, cross-attention, financial risk modeling, loan default prediction

## Abstract

Credit risk assessment is a core component of financial decision-making. This study develops an explainable machine learning framework for modeling loan approval decisions on heterogeneous tabular data, centered on a Cross-Attentional Tabular Transformer that applies bidirectional cross-attention between numerical and categorical feature groups. The prediction target is historical loan-approval status, treated as a proxy for, not a direct measure of, borrower default risk; a supplementary validation on a dataset with an authentic default label is also reported. Class imbalance is addressed through focal loss, and post hoc interpretability is provided through SHAP analysis. Three classifiers, Random Forest, Gradient Boosting, and the proposed transformer, are evaluated on a 5000-sample credit dataset using accuracy, precision, recall, F1-score, ROC-AUC, and average precision. Gradient Boosting achieves the best performance (accuracy 0.9640, F1-score 0.9189), with Random Forest comparable; the proposed transformer reaches 0.9530 accuracy and 0.8949 F1, without surpassing the ensembles and at substantially higher computational cost. A five-split robustness comparison additionally evaluates XGBoost, LightGBM, CatBoost, and calibrated logistic regression: all three Gradient-Boosting variants and both classical ensembles exceed the transformer’s performance on every metric, while calibrated logistic regression does not. The evaluated baseline set excludes deep tabular architectures such as TabNet, FT-Transformer, SAINT, and TabPFN-style methods. Across the three primary classifiers, SHAP identifies credit score, employment status, and income as the dominant features, consistent with domain expectations. The results characterize the observed performance–efficiency trade-off between ensemble methods and attention-based tabular learning under the evaluated data conditions.

## 1. Introduction

### 1.1. Research Background and Motivation

Credit risk assessment constitutes a core component of modern financial decision-making, with direct implications for loan approval, credit allocation, portfolio quality, and institutional resilience [1]. For banks, consumer finance providers, and fintech platforms, the ability to distinguish between low-risk and high-risk applicants is essential to mitigating default losses, improving capital utilization, and maintaining consistency in lending practice [2]. As financial services continue to evolve toward data-driven operation, credit evaluation has gradually shifted from rule-based screening and expert judgment to predictive modeling frameworks capable of extracting decision patterns from historical borrower information.

Conventional credit risk assessment has long been dominated by statistical and machine learning approaches tailored to structured data [3]. Logistic regression, Decision Trees, Random Forests, and Gradient Boosting methods remain widely adopted because they offer solid predictive performance, mature deployment pipelines, and comparatively interpretable decision logic. Among them, ensemble tree models have shown particular strength in tabular classification tasks and therefore occupy a central position in credit scoring practice. Nevertheless, such methods remain constrained in their ability to represent complex dependence structures when borrower risk is governed by heterogeneous, nonlinear, and interaction-intensive relationships. In practical lending scenarios, repayment risk is rarely determined by isolated variables; rather, it emerges from coupled effects among income, loan amount, credit score, work experience, employment type, and other demographic or financial indicators. These dependencies are often only partially represented through manually designed interaction terms or recursive partitioning mechanisms.

Recent progress in deep learning for tabular data has introduced a new modeling paradigm for structured financial prediction [4]. Transformer-based architectures, initially developed for sequence representation learning, have demonstrated increasing promise in tabular applications due to their ability to model high-order feature interactions through attention mechanisms. Relative to conventional approaches, deep tabular models provide a more flexible representation space in which latent dependencies among mixed numerical and categorical variables can be learned directly from data. This property is particularly relevant to credit risk assessment, where the predictive contribution of individual variables is frequently contingent upon broader contextual combinations that are difficult to specify in advance. The demand for intelligent and adaptive risk modeling systems therefore motivates a more systematic examination of attention-based learning in credit assessment tasks.

### 1.2. Research Problem and Challenges

Although credit risk assessment is commonly formulated as a binary classification problem, its practical realization is complicated by several characteristics intrinsic to financial tabular data. A central difficulty lies in the coexistence of heterogeneous feature types. Credit datasets typically combine continuous numerical variables, such as age, income, loan amount, credit score, and years of experience, with categorical descriptors such as education level, employment type, and geographic location. These variables differ substantially in scale, distribution, semantics, and representation requirements, making unified modeling nontrivial.

Another challenge arises from class imbalance. In many lending scenarios, low-risk or approved cases substantially outnumber adverse outcomes, causing standard optimization objectives to favor the majority class. Under such conditions, seemingly strong overall accuracy may conceal inadequate identification of risky applicants. Because the financial cost associated with misclassifying a high-risk borrower is markedly higher than that of rejecting a creditworthy one, imbalance handling is not merely a statistical refinement but a substantive modeling requirement in credit prediction.

The predictive structure of credit data further increases the difficulty of the task. Borrower risk is seldom governed by simple additive relationships among independent variables. The effect of a specific loan amount, for example, may depend strongly on income level, prior credit standing, employment category, or experience-related repayment capacity. Likewise, identical credit scores may imply different levels of default exposure under different financial profiles. Such context-dependent and nonlinear interactions are not always captured effectively by shallow models or manually engineered combinations of variables, thereby creating a strong demand for representation mechanisms capable of learning complex feature dependencies in a data-adaptive manner.

An additional challenge concerns model transparency. Financial institutions require more than accurate predictions; they also require interpretable reasoning regarding how a model reaches its conclusions, which variables dominate the decision process, and whether the resulting patterns remain consistent with domain expectations. Models that offer strong predictive performance but limited interpretability face practical barriers in deployment because decision processes in credit evaluation must remain auditable, inspectable, and suitable for expert review. Credit risk modeling therefore entails a dual objective: achieving strong predictive discrimination while preserving interpretive accessibility.

These considerations define the core problem addressed in this study: the development of a credit risk assessment framework capable of modeling heterogeneous and interaction-intensive tabular data, accommodating skewed class distributions, and providing transparent decision support within a unified predictive architecture.

### 1.3. Main Contributions

This study develops an explainable deep learning framework for credit risk assessment centered on attention-based tabular representation. The main contributions are summarized as follows.

(1) A Cross-Attentional Tabular Transformer is designed for credit risk assessment on heterogeneous tabular data.
(2) An imbalance-aware learning strategy is introduced to improve minority-class recognition under skewed class distributions.
(3) SHAP, a model-agnostic post hoc explanation technique applicable to all evaluated classifiers, is used to provide global and local interpretations and to assess whether the learned decision logic aligns with domain expectations.
(4) A comparative evaluation is performed against Random Forest, Gradient Boosting, and, in an extended five-split robustness comparison, XGBoost, LightGBM, CatBoost, and calibrated logistic regression. Ablation studies isolate the contributions of the cross-attention mechanism and engineered ratio features. Deep tabular architectures such as TabNet, FT-Transformer, SAINT, and TabPFN-style small-data methods fall outside the evaluated comparison set.

This methodological study examines attention-based representation for heterogeneous tabular credit data using a structured benchmark dataset of 5000 loan application records. The analysis focuses on representational properties, interpretability characteristics, and performance trade-offs under controlled experimental conditions. Applicability to larger-scale, institution-specific, or geographically diverse credit environments requires further empirical validation. The primary experiments use historical loan-approval status as a proxy for borrower default risk, while Section 4.7 reports a supplementary validation on a dataset with an authentic default label.

### 1.4. Organization of the Paper

The remainder of this paper is organized as follows.

Section 2 reviews the related literature on traditional credit risk assessment, deep learning methods for tabular financial data, and explainable artificial intelligence in risk modeling.

Section 3 presents the proposed methodology, including the overall framework, dataset preprocessing, feature representation strategy, Cross-Attentional Tabular Transformer, imbalance-aware optimization, and explainability analysis.

Section 4 reports the experimental results, including baseline comparisons, ablation analysis, sensitivity analysis, interpretability findings, and a supplementary validation on a real-default-labeled dataset.

Section 5 integrates the discussion of the main findings, practical implications, study limitations, and concluding remarks.

## 2. Related Works

### 2.1. Traditional Credit Risk Assessment Methods

Credit risk assessment has long been dominated by statistical learning and conventional machine learning methods designed for structured financial data [5]. Among them, logistic regression remains one of the most widely adopted approaches in banking and consumer finance because of its clear probabilistic interpretation, stable training behavior, and relatively transparent decision logic [6]. Its linear formulation allows risk factors to be directly associated with estimated default tendencies, which makes it particularly suitable for scorecard construction and risk screening tasks. Nevertheless, its modeling capacity is inherently limited when the relationships among borrower attributes exhibit strong nonlinearity, high-order dependence, or context-specific interactions. In such cases, extensive manual feature engineering is usually required to achieve competitive predictive performance.

Tree-based models provide a more flexible alternative by capturing nonlinear decision boundaries through recursive partitioning. Decision Trees are intuitive and easy to interpret, but their predictive stability is often weakened by high variance and sensitivity to data perturbation. Random Forest addresses part of this limitation by aggregating multiple trees through bootstrap sampling and feature randomness, thereby improving robustness and generalization. In credit risk tasks, Random Forest has shown strong performance because it can accommodate mixed feature types and capture complex variable relationships without heavy preprocessing [7]. Even so, ensemble averaging improves stability at the cost of reduced transparency, and the model still lacks an explicit mechanism for learning structured feature interactions beyond hierarchical splitting behavior.

Gradient-Boosting methods further advance predictive performance by sequentially correcting the residual errors of prior learners. Algorithms such as Gradient-Boosting Decision Tree, XGBoost, LightGBM, and CatBoost have become highly competitive in financial risk modeling due to their strong fitting ability, efficient optimization, and adaptability to tabular data. These methods are particularly effective when predictive patterns arise from nonlinear combinations of borrower characteristics, making them a frequent benchmark in credit scoring and default prediction studies [8]. However, their superiority in tabular accuracy does not eliminate several limitations relevant to modern financial applications. Their interaction modeling remains implicit within tree ensembles rather than representation-driven, their outputs can become difficult to interpret at a fine-grained level when model complexity increases, and their extensibility to more heterogeneous or multi-source learning settings is comparatively limited.

Taken together, traditional credit risk assessment methods remain highly effective and operationally mature, especially for structured lending data. Their limitations emerge primarily when the task requires richer representation of feature interactions, stronger adaptability to heterogeneous data structures, or tighter integration between predictive modeling and post hoc interpretability analysis.

### 2.2. Deep Learning for Tabular Data

The growing interest in deep learning for structured data has motivated a broad line of research aimed at overcoming the representational limits of conventional tabular models [9]. Early approaches were largely based on multilayer perceptrons, which treat all input variables as dense vectors and learn nonlinear mappings through stacked fully connected layers [10]. MLP-based models offer a unified differentiable framework and can approximate complex decision boundaries, but they often struggle to outperform strong tree-based baselines on tabular tasks. One reason is that standard MLPs do not explicitly exploit the structural heterogeneity of tabular variables, and their ability to capture informative cross-feature interactions depends heavily on network depth, feature preprocessing, and data scale.

To address this limitation, a number of deep tabular architectures have introduced more specialized design principles. Deep and Cross Network explicitly models bounded-order feature interactions through cross layers, thereby improving learning efficiency in settings where structured variable combinations are central to prediction [11]. TabNet employs sequential attentive feature selection, enabling the model to focus adaptively on salient variables at different decision stages while preserving a degree of sparsity and interpretability [12]. TabTransformer introduces contextual embedding of categorical variables through self-attention, making it possible to learn richer inter-feature dependencies than conventional embedding–MLP pipelines. FT-Transformer further generalizes transformer-based tabular learning by tokenizing both numerical and categorical features and applying attention-based representation learning in a more unified manner.

These developments have demonstrated that deep tabular learning can provide a flexible alternative to ensemble trees, particularly when the objective extends beyond static classification to broader representation learning, modular integration, or multi-modal expansion [13]. Even so, several limitations remain visible in the current literature. Many models emphasize general predictive performance while paying less attention to explicit interaction mechanisms tailored to heterogeneous financial variables. Some architectures are effective at learning latent dependencies, yet they do not sufficiently distinguish between different types of relational structure among numerical and categorical attributes. Others incorporate interaction modeling, but do not integrate such design with imbalance-aware optimization or model-level explainability in a unified framework. As a result, existing deep tabular approaches often remain incomplete when evaluated against the combined requirements of credit risk assessment, where explicit interaction learning, heterogeneous feature representation, and interpretable decision support are all of practical importance.

### 2.3. Explainable AI in Financial Risk Modeling

Explainable artificial intelligence has become increasingly important in financial risk modeling because predictive accuracy alone is insufficient for high-stakes decision systems [14]. In credit assessment, model outputs may affect loan approval, pricing, and portfolio exposure, which in turn creates strong demand for transparent reasoning and traceable decision support [15]. Financial institutions must be able to identify which variables drive model predictions, whether the learned patterns are consistent with domain expectations, and how individual applicant assessments can be justified under expert review. This requirement has made explainability a central rather than auxiliary component of intelligent credit evaluation.

Among widely used post hoc interpretation tools, SHAP has received substantial attention because it provides a theoretically grounded mechanism for feature attribution at both the global and local levels [16]. By quantifying the marginal contribution of each feature to the prediction outcome, SHAP enables portfolio-level ranking of important variables as well as sample-level explanation of individual risk decisions. LIME offers another local explanation strategy by approximating complex models with interpretable surrogate models in the neighborhood of a target instance. Partial Dependence analysis is also commonly employed to reveal the marginal influence of selected variables on model output, thereby supporting examination of nonlinear response patterns and feature-effect trends. These methods have become valuable tools for interpreting black-box models in practical credit risk applications.

Despite these advances, explainability in financial modeling remains challenging. Post hoc interpretation does not fully eliminate the opacity of complex predictive architectures, particularly when multiple interacting variables jointly influence the model output [17]. In real credit approval settings, institutions are concerned not only with feature importance ranking, but also with whether the explanation is stable, decision-relevant, and aligned with financial reasoning. This concern becomes more prominent when black-box models are deployed in scenarios requiring consistency, auditability, and operational trust. Consequently, the practical value of explainable AI in credit risk assessment lies not merely in producing visual explanations, but in embedding interpretability into the broader modeling pipeline so that predictive performance and decision transparency can be considered jointly.

### 2.4. Summary and Research Positioning

Existing studies on credit risk assessment generally follow two dominant lines. One is centered on traditional machine learning methods, including logistic regression, Random Forest, and Gradient Boosting variants, which remain strong baselines for structured financial prediction because of their solid performance, engineering maturity, and practical usability. The other is oriented toward deep tabular learning, where models such as TabNet, TabTransformer, FT-Transformer, and Deep and Cross Network have expanded the representational capacity of credit risk modeling by introducing nonlinear transformation and attention-based dependency learning. Taken together, these studies have substantially advanced automated credit assessment, yet they also reveal a clear separation between conventional predictive robustness and emerging representation learning flexibility.

Despite their effectiveness, traditional machine learning methods remain limited in their ability to model complex and heterogeneous feature interactions in a more expressive manner. Their predictive logic is largely built on linear assumptions, recursive partitioning, or ensemble aggregation, which often captures interaction effects only implicitly. Deep tabular models alleviate part of this limitation by learning latent dependencies directly from data, but many existing architectures are designed as general-purpose solutions rather than methods specifically tailored to the structural characteristics of credit data. In particular, the explicit modeling of cross-type relationships among heterogeneous borrower attributes remains insufficiently explored, even though such dependencies are central to credit risk formation.

Another important limitation in the current literature concerns the weak integration of imbalance-aware learning and model explainability. In practical credit datasets, minority-risk identification is often more important than aggregate classification accuracy, yet class imbalance is frequently treated as an auxiliary issue rather than a core modeling objective. At the same time, interpretability is commonly appended after model development rather than considered as part of the methodological design. This fragmented treatment is especially problematic in financial applications, where predictive performance, risk sensitivity, and decision transparency must be considered jointly. As a result, a unified framework that simultaneously addresses heterogeneous tabular representation, skewed class distributions, and interpretable credit decision support remains underdeveloped.

The present study addresses this gap through an integrated framework for intelligent credit risk assessment that combines cross-attentional tabular representation learning, imbalance-aware optimization, and SHAP-based explainability analysis. The framework connects traditional financial risk modeling with recent deep tabular learning while aligning model design with the practical requirements of credit evaluation.

As shown in Table 1, existing studies on credit risk prediction have mainly evolved along several lines, including review-oriented analyses, ensemble-based predictive modeling, sampling strategies for imbalanced data, explainable machine learning, and specialized model design. Prior studies have made important progress in improving predictive performance, enhancing model transparency, and addressing data imbalance. However, most existing works emphasize only one or two aspects at a time, such as accuracy improvement, resampling effectiveness, or post hoc interpretation. In contrast, this work integrates imbalance handling, cost-sensitive learning, predictive performance, and interpretability into a unified framework, thereby providing a more decision-relevant and financially meaningful solution for credit risk prediction.

## 3. Materials and Methods

### 3.1. Dataset and Overall Framework

#### 3.1.1. Problem Definition

This study considers credit risk assessment as a supervised binary classification task defined on heterogeneous tabular data. Let the raw dataset be denoted by

$$D\;=\;{\left\{\left(x_{i},y_{i}\right)\right\}}_{i=1}^{N}$$

where N is the number of loan application records, $x_{i}$ denotes the feature vector of the i-th applicant, and $y_{i}\in \{0,1\}$ is the corresponding binary credit outcome. In the present study, the dataset contains N=5000 samples. Each applicant record is composed of numerical and categorical attributes, including age, income, loan amount, credit score, years of experience, gender, education, city, and employment type. The target variable is the loan approval outcome.

The input space is heterogeneous by construction. Let

$$x_{i}\;=\;\left[x_{i}^{\left(n\right)},x_{i}^{\left(c\right)}\right]$$

where $x_{i}^{\left(n\right)}\in R^{d_{n}}$ denotes the numerical feature subvector and $x_{i}^{\left(c\right)}\in R^{d_{c}}$ denotes the categorical feature subvector after appropriate encoding. In this dataset, $d_{n}\;=\;$ 5 and $d_{c}\;=\;\;4$ at the raw feature level. The predictive objective is to learn a scoring function

$$f_{\theta }:X\to [0,\;1]$$

that estimates the conditional probability of the positive class,

$${\hat{p}}_{i}\;=\;f_{\theta }\left(x_{i}\right)\;\approx \;P\left(y_{i}\;=\;1|x_{i}\right)$$

The final class prediction is obtained by applying a decision threshold τ to the estimated probability:

$${\hat{y}}_{i}\;=\;\left\{\begin{array}{cc} 1, & {\hat{p}}_{i}\;\geq \;\tau \\ 0, & {\hat{p}}_{i}\;<\;\tau \end{array}\right.\;$$

where τ denotes the decision threshold.

The methodological objective is therefore not limited to binary discrimination alone. A practically meaningful credit risk model should satisfy three simultaneous requirements: adequate predictive performance on structured applicant data, sufficient capacity to model nonlinear and cross-feature dependencies, and interpretability of the resulting decisions in a financial context. The framework developed in this study is constructed in accordance with these requirements.

#### 3.1.2. Overall Pipeline

The methodological workflow is organized as a complete learning-and-interpretation pipeline extending from raw credit records to explainable model outputs. Let the raw dataset be written as

$$D_{\mathrm{raw}\;}\;=\;{\left\{\left(x_{i}^{\mathrm{raw}},y_{i}\right)\right\}}_{i=1}^{N}$$

where $x_{i}^{\mathrm{raw}\;}$ contains the original borrower attributes. Because the raw records contain mixed feature types and missing entries, a preprocessing function

Φ(·)

is introduced to transform the original data into model-ready representations:

$$z_{i}\;=\;\Phi \left(x_{i}^{\mathrm{raw}}\right)$$

The transformed sample $z_{i}$ is then used as the effective input for downstream classifiers.

The preprocessing stage includes missing-value treatment, categorical encoding, numerical normalization, and dataset partitioning. For numerical variables, the transformation can be expressed as

$$z_{\mathrm{ij}}^{\left(n\right)}\;=\;\frac{x_{\mathrm{ij}}^{\left(n\right)}-\mu_{j}}{\sigma_{j}}$$

where $\mu_{j}$ and $\sigma_{j}$ are the mean and standard deviation of the j-th numerical feature estimated from the training set. For categorical variables, model-specific encoding is employed. In the traditional machine learning branch, categorical attributes are converted into one-hot vectors, resulting in processed datasets of dimensions 4000×19 for training and 1000×19 for testing. Accordingly, the processed dataset can be denoted by

$$D_{\mathrm{proc}\;}\;=\;{\left\{\left(z_{i},y_{i}\right)\right\}}_{i=1}^{N}$$

After preprocessing, the dataset is partitioned into disjoint training and testing subsets:

$$D_{\mathrm{proc}\;}\;=\;D_{\mathrm{train}\;}\cup D_{\mathrm{test}\;},\;D_{\mathrm{train}\;}\cap D_{\mathrm{test}\;}\;=\;\emptyset$$

with $\left|D_{\mathrm{train}\;}\right|\;=\;4000$ and $\left|D_{\mathrm{test}\;}\right|\;=\;1000$. The training subset is used for model fitting and parameter optimization, whereas the testing subset is reserved for out-of-sample performance evaluation. Let $f_{\theta }^{\left(m\right)}$ denote the m-th classifier under consideration. Model training is then formulated as

$$\theta^{(m)*}\;=\;\arg\min_{\theta^{\left(m\right)}}\;L^{\left(m\right)}\left(D_{\mathrm{train}\;};\theta^{\left(m\right)}\right)$$

where $L^{\left(m\right)}$ denotes the corresponding training objective. The trained classifier produces probability estimates and class predictions on the test set, which are further assessed through multiple evaluation metrics.

The final component of the pipeline is post hoc explanation analysis. Given a trained predictor $f_{\theta^{*}}$, an explainability operator

Ψ(·)

is introduced to quantify the contribution of each feature to the prediction:

$$s_{i}\;=\;\Psi \left(f_{\theta^{*}},z_{i}\right)$$

where $s_{i}$ denotes the explanation vector associated with the i-th sample. This step enables the transition from predictive output to decision interpretation, thereby linking model performance with risk-factor attribution at both the global and local levels.

#### 3.1.3. Technical Route

The technical route of the proposed framework is centered on three tightly coupled components: heterogeneous feature representation through cross-attentional modeling, imbalance-aware optimization for minority-class recognition, and explanation-oriented analysis for transparent credit decision support. The purpose of this design is to ensure that predictive modeling, risk sensitivity, and interpretability are treated as parts of a single methodological system rather than as loosely connected post hoc additions.

For heterogeneous tabular inputs, the core modeling problem lies in how to represent and fuse numerical and categorical borrower attributes while preserving their interaction structure. Let

$$h_{i}^{\left(n\right)}\;=\;g_{n}\left(x_{i}^{\left(n\right)}\right),\;h_{i}^{\left(c\right)}\;=\;g_{c}\left(x_{i}^{\left(c\right)}\right)$$

where $g_{n}(\cdot )$ and $g_{c}(\cdot )$ denote the representation mappings for numerical and categorical features, respectively. The proposed framework introduces a cross-attention mechanism to model dependency exchange between these two feature groups. In generic form, the cross-attention operation can be written as

$$\mathrm{Attn}(Q,K,V)\;=\;\mathrm{softmax}\left(\frac{{\mathrm{QK}}^{⊤}}{\sqrt{d}}\right)V$$

where Q, K, and V are the query, key, and value matrices derived from heterogeneous feature embeddings, and d is the feature dimension used for scaling. This mechanism enables one feature group to attend to the informative patterns of another, thereby improving the representation of nonlinear cross-feature dependencies that are central to credit risk formation.

The optimization stage is designed to remain sensitive to asymmetric class distributions. Let $y_{i}\in \{0,1\}$ and let ${\hat{p}}_{i}$ denote the predicted probability for the positive class. Under standard binary cross-entropy, the loss can be written as

$$L_{\mathrm{BCE}}\;=\;-\frac{1}{N}\sum_{i=1}^{N}\;\left[y_{i}\log\left({\hat{p}}_{i}\right)\;+\;\left(1-y_{i}\right)\log\left(1-{\hat{p}}_{i}\right)\right]$$

Since this objective may bias learning toward the majority class under skewed distributions, an imbalance-aware variant is incorporated in the proposed framework. In its general weighted form,

$$L_{\mathrm{imb}}\;=\;-\frac{1}{N}\sum_{i=1}^{N}\;\left[\alpha_{1}y_{i}\log\left({\hat{p}}_{i}\right)\;+\;\alpha_{0}\left(1-y_{i}\right)\log\left(1-{\hat{p}}_{i}\right)\right]$$

where $\alpha_{1}$ and $\alpha_{0}$ are class-dependent weighting coefficients. When focal-style modulation is adopted, the objective can be further extended as

$$L_{\mathrm{focal}\;}\;=\;-\frac{1}{N}\sum_{i=1}^{N}\;\left[\alpha_{1}y_{i}{\left(1-{\hat{p}}_{i}\right)}^{\gamma }\log\left({\hat{p}}_{i}\right)\;+\;\alpha_{0}\left(1-y_{i}\right){\hat{p}}_{i}^{\gamma }\log\left(1-{\hat{p}}_{i}\right)\right]$$

where γ controls the emphasis placed on hard-to-classify samples. This formulation improves the alignment between model training and the practical requirement of correctly identifying minority-class (Approved) applicants.

Interpretability is incorporated through a feature-attribution mechanism applied after model fitting. For a trained predictor $f_{\theta^{*}}$, the contribution of the j-th feature to the prediction of sample i is denoted by $\phi_{\mathrm{ij}}$. The explanation vector

$$\phi_{i}\;=\;\left[\phi_{i1},\phi_{i2},\;\ldots ,\;\phi_{\mathrm{id}}\right]$$

provides an additive decomposition of the model output around a reference value:

$$f_{\theta^{*}}\left(z_{i}\right)\;=\;\phi_{0}\;+\;\sum_{j=1}^{d}\;\phi_{\mathrm{ij}}$$

where $\phi_{0}$ denotes the baseline prediction and d is the number of processed input dimensions. This formulation makes it possible to examine dominant predictors at the population level and to interpret the specific reasons behind an individual credit decision. The resulting technical route therefore links representation learning, imbalance-aware prediction, and decision explanation into a coherent methodological framework for intelligent credit risk assessment.

Ancillary feature-screening results, model hyperparameter configurations, and per-class performance values are provided in Appendix A.

### 3.2. Dataset and Data Preprocessing

#### 3.2.1. Dataset Description

The empirical study was conducted on a structured credit risk dataset containing 5000 loan application records. Each record represents an individual applicant and includes demographic, financial, and employment-related information together with a binary target label indicating the credit assessment outcome. The raw feature space comprises five numerical variables, namely Age, Income, LoanAmount, CreditScore, and YearsExperience, as well as four categorical variables, namely Gender, Education, City, and EmploymentType. The target variable is LoanApproved.

Table 2 summarizes the structure of the raw dataset and shows that the prediction task is defined on a compact but heterogeneous feature space composed of financial, demographic, and employment-related variables. Missing values are confined to Income, CreditScore, and Education, while all remaining fields are complete. This pattern indicates that the dataset quality is generally stable and that the missing-data problem is localized rather than pervasive, which justifies the adoption of conventional yet robust imputation strategies in the preprocessing stage.

The dataset exhibits two characteristics that are directly relevant to model construction. The first is feature heterogeneity, since the input space contains both continuous and categorical variables with different statistical properties and semantic roles. The second is incomplete information in several original fields. Missing values are present in Income, CreditScore, and Education, whereas the remaining variables are complete. In addition, the class distribution is imbalanced, with the negative class accounting for the majority of samples. This distributional skew implies that a classifier trained without explicit imbalance consideration may favor overall accuracy while remaining insufficiently sensitive to minority-class patterns.

**Figure 1:**
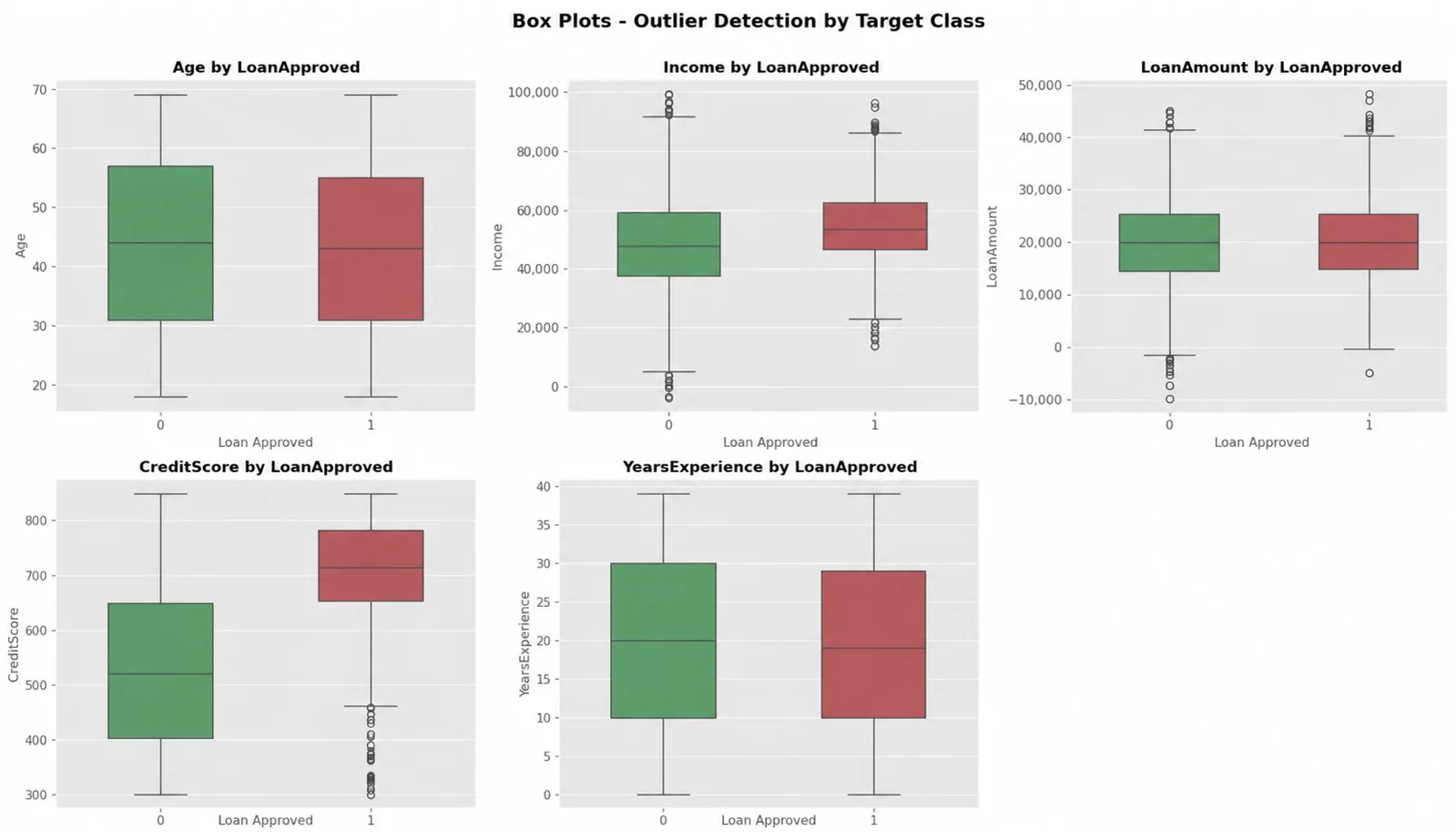
Box plots of the numerical variables grouped by the target class.

The dataset is a structured benchmark collection for supervised learning on heterogeneous tabular data rather than a sample from a specific lending institution or regional financial market. Its 5000 records are modest relative to annual commercial lending volumes, and the four represented cities do not capture the full diversity of borrower profiles across economic environments. The income range, including the extreme observations in Figure 1, extends from approximately −5 K to 100 K, and the uniform city distribution indicates a population-level benchmark design. The resulting findings characterize model design choices and representational trade-offs under this dataset configuration.

Table 3 provides a descriptive summary of the numerical variables in the raw dataset. The statistics indicate that the numerical predictors span substantially different value ranges, especially for Income, LoanAmount, and CreditScore, which supports the need for feature normalization before model training. The distributional spread of CreditScore and Income further suggests that these variables carry substantial discriminatory potential, whereas Age and YearsExperience appear more likely to serve as supporting variables whose contribution may depend on interactions with other financial attributes.

Figure 1 reveals that the numerical variables differ substantially in their class-discriminative behavior. CreditScore shows the clearest separation between the Approved and Not Approved groups, with markedly higher central tendency in the Approved class. Income also exhibits a visible upward shift for approved samples, although the overlap between classes remains non-negligible. By contrast, Age and YearsExperience present much stronger overlap, while LoanAmount alone does not provide strong class separation. These patterns indicate that the predictive structure of the task is more strongly related to credit quality and repayment capacity than to simple demographic or exposure magnitude variables considered in isolation.

**Figure 2:**
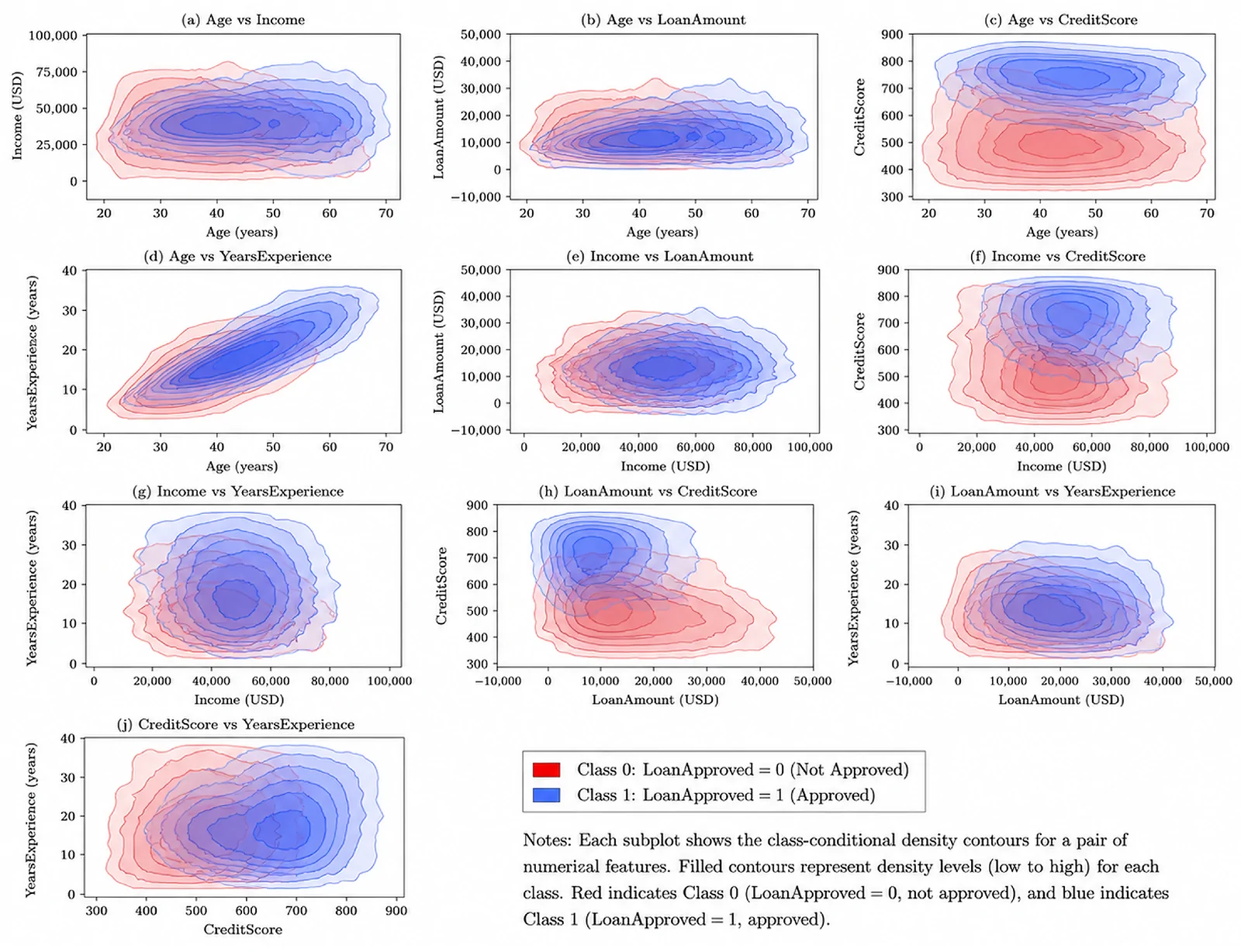
Class-conditional density contour plots for pairwise combinations of the numerical variables. Red contours correspond to the Not Approved class (class 0, LoanApproved = 0), and blue contours correspond to the Approved class (class 1, LoanApproved = 1). Filled contours represent density levels from low (outer) to high (inner) for each class.

Figure 2 visualizes the class-conditional joint distributions of the numerical variables through pairwise density contours. The substantial overlap between the two class distributions in most variable pairs confirms that the class separation cannot be described by simple univariate thresholds alone, and that the decision boundary is unlikely to be linear or low-dimensional. The contour concentrations differ most clearly for pairs involving CreditScore and Income, where the Approved class tends to shift toward higher values relative to the Not Approved class. By contrast, pairs involving Age and YearsExperience show considerably more distributional overlap between the two classes, indicating weaker class-discriminative structure in those dimensions. These patterns suggest that the predictive mechanism is driven less by isolated marginal effects than by conditional relationships among key financial variables, which provides a direct motivation for interaction-aware modeling.

**Figure 3:**
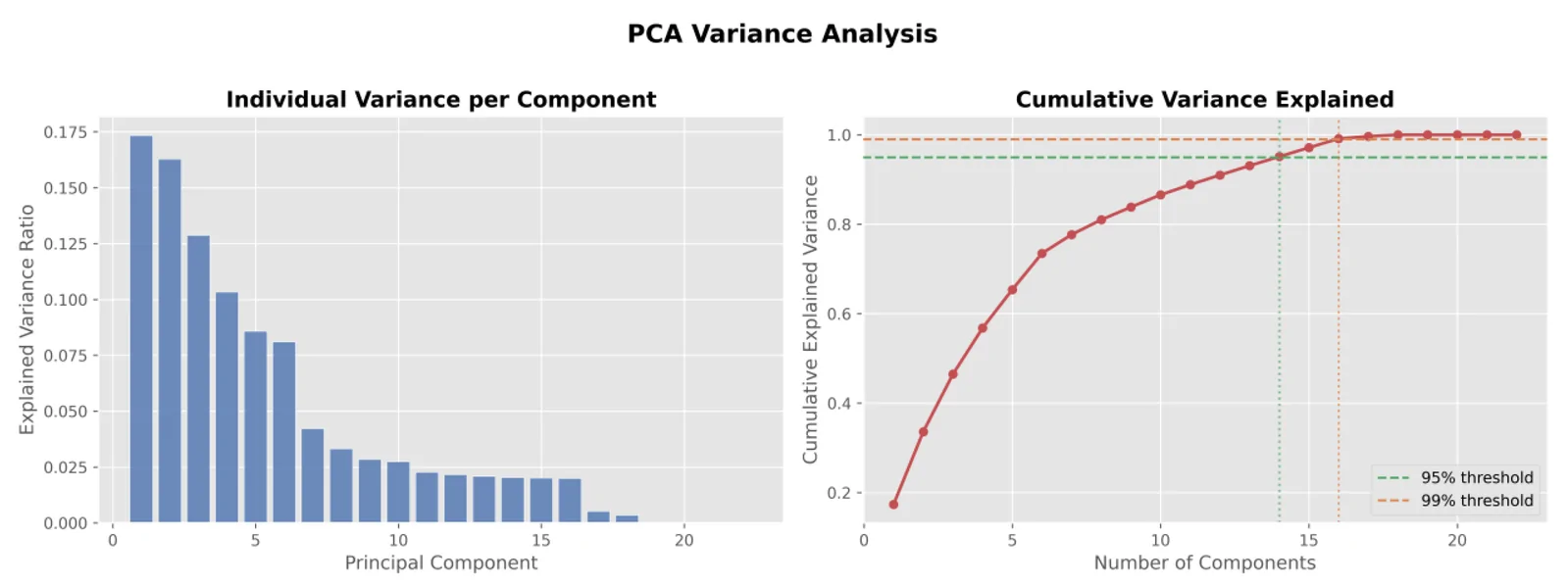
PCA variance analysis of the processed training data. The vertical dashed lines mark the minimum numbers of principal components required to reach 95% (green) and 99% (orange) cumulative explained variance.

Figure 3 shows that the variance of the processed feature space is distributed across multiple principal components rather than being concentrated in only a few dominant directions. A relatively large number of components is required to retain high cumulative variance, which indicates that the data structure is not easily compressible into a very low-dimensional representation. This observation is consistent with the overlap seen in the pairwise scatter plots and suggests that the credit prediction task contains distributed and interaction-dependent information. It therefore provides additional support for the use of models capable of capturing nonlinear dependencies in a higher-dimensional tabular feature space.

From the perspective of supervised learning, the raw data can be regarded as borrower-level observations composed of mixed-type predictors and binary labels. The methodological role of preprocessing is therefore to transform these original records into model-ready representations while preserving the predictive information contained in the financial and demographic attributes.

#### 3.2.2. Data Cleaning and Encoding

The preprocessing steps described in this subsection follow standard practices for heterogeneous tabular data; they are summarized briefly for reproducibility rather than presented as a methodological contribution, since the design choices specific to this work concern the model architecture and the domain-informed feature construction described in Section 3.3.

Missing numerical entries are imputed with the training-set median of the corresponding variable, and missing categorical entries are imputed with the most frequent category, with both statistics estimated from the training data only. The imputed records are then encoded according to the preprocessing operator Φ(·) introduced in Section 3.1.2, whose concrete form depends on the modeling branch. For the conventional machine learning branch, categorical variables are one-hot encoded and concatenated with the standardized original numerical variables, yielding 18 predictive variables before feature engineering (five numerical features and thirteen encoded categorical indicators). After the four engineered continuous features described in Section 3.3.1 are appended, the final conventional-model input contains 22 predictive variables. For the transformer-based branch, categorical variables are instead preserved as integer category indices for subsequent embedding-based representation learning, since attention-based embedding layers require discrete category identities rather than one-hot-expanded indicators. Both branches originate from the same raw borrower records but produce inputs adapted to the representational assumptions of the corresponding model family.

#### 3.2.3. Data Splitting and Normalization

Following data cleaning and encoding, the processed data are divided into training and testing subsets according to an 80%/20% split. This yields 4000 samples for training and 1000 samples for testing. The same partition preserves the imbalanced class structure observed in the original dataset, thereby ensuring that model evaluation is conducted under a realistic credit prediction setting rather than on an artificially balanced sample space.

Table 4 shows that the imbalanced class structure of the original dataset is preserved after train–test partitioning. The class proportions remain nearly unchanged across the raw, training, and testing subsets, indicating that the split does not distort the original label distribution. This preservation is methodologically important because it ensures that the subsequent evaluation reflects the actual class skew of the credit risk problem rather than an artificially balanced setting.

Numerical variables are standardized using the transformation already defined in Section 3.1.2, with the mean and standard deviation estimated from the training set only so that no test-set information leaks into preprocessing.

The final processed data used for the traditional-learning branch therefore consist of five standardized original numerical variables, four engineered continuous variables, thirteen one-hot encoded categorical indicators, and the associated binary target label, whereas the transformer-oriented branch retains the same underlying applicant information in a form compatible with embedding-based attention modeling.

Table 5 clarifies the final structure of the processed data used by the traditional machine learning branch. Standardization and one-hot encoding first produce an 18-dimensional representation of the original variables; appending the four engineered continuous features then yields 22 predictive variables in the final model input. This representation is well aligned with the input assumptions of conventional tabular classifiers and also makes clear that the processed data used for baseline models differ structurally from the embedding-oriented representation required by the transformer branch.

### 3.3. Feature Engineering and Representation

#### 3.3.1. Domain-Informed Feature Construction

The predictive structure of credit risk data is rarely determined by isolated variables alone. In practical lending scenarios, risk often emerges from the interaction between repayment capacity, credit quality, requested exposure, and employment-related stability. For this reason, feature construction in this study is not restricted to direct use of the original borrower attributes, but is extended through domain-informed transformations intended to strengthen the representation of financially meaningful relationships.

A central consideration in this process is the ratio relationship between requested credit and available repayment capacity. Let $I_{i}$ denote the income of applicant i, and let $L_{i}$ denote the requested loan amount. A basic exposure indicator is then defined as the loan-to-income ratio,

$$r_{i}^{\left(\mathrm{LTI}\right)}\;=\;\frac{L_{i}}{I_{i}\;+\;1}$$

where the denominator is offset by a constant of one, the value used throughout this study, to avoid numerical instability when income is close to zero. This variable provides a normalized measure of financial burden and is more informative than the loan amount alone when applicants differ substantially in income level.

A second interaction is constructed to reflect the relationship between credit quality and accumulated work experience. Let $C_{i}$ denote the credit score and $E_{i}$ denote the years of experience. An experience-adjusted credit indicator is written as

$$r_{i}^{\left(\mathrm{CE}\right)}\;=\;\frac{C_{i}}{E_{i}\;+\;1}$$

where the same unit offset is applied because $E_{i}$ can equal zero for applicants with no prior work experience, which would otherwise leave the ratio undefined. This transformation is intended to capture a relative notion of credit standing under different experience levels. While credit score and work experience are individually informative, their joint relation may reveal distinct borrower profiles that are not fully expressed through additive modeling alone.

A third interaction expresses loan exposure relative to credit quality directly, rather than the credit-quality-normalized relationship considered above. A risk index is defined as

$$r_{i}^{\left(\mathrm{RI}\right)}\;=\;\frac{L_{i}}{C_{i}\;+\;1}$$

This variable increases with the requested loan amount and decreases with credit score, so that, in contrast to the indicators above, a higher value corresponds to greater exposure relative to demonstrated creditworthiness. In credit evaluation, the same requested loan amount may represent different levels of risk depending on the applicant’s credit standing; this ratio provides a compact representation of that conditional exposure.

In addition to these three ratio features, an income-per-age indicator is constructed as

$$r_{i}^{\left(\mathrm{IA}\right)}\;=\;\frac{I_{i}}{A_{i}\;+\;1}$$

where $A_{i}$ denotes the applicant’s age. This variable normalizes income by age to capture relative earning position independent of absolute income level. It is included in the preliminary feature analysis and explainability results reported in Section 3.3.3 and Section 4.6, but it is not one of the three ratio features toggled in the feature ablation study reported in Section 4.4. The resulting feature engineering design therefore emphasizes interaction-aware construction grounded in financial interpretation rather than indiscriminate variable expansion.

#### 3.3.2. Feature Representation for Tabular Learning

After feature construction, the predictor space is organized according to the representational requirements of the downstream models. Since the study involves both conventional machine learning methods and an attention-based tabular architecture, feature representation is designed in a model-consistent manner rather than forced into a single encoding scheme.

For the traditional learning branch, the numerical variables and the derived ratio-based indicators are represented as continuous inputs after normalization, whereas the categorical variables are transformed into one-hot encoded binary vectors. The final feature vector can be written in concatenated form as

$$z_{i}=\left[{\tilde{x}}_{i}^{\left(n\right)},r_{i},o_{i}^{\left(c\right)}\right]$$

where ${\tilde{x}}_{i}^{\left(n\right)}$ denotes the standardized original numerical features, $r_{i}$ denotes the set of engineered interaction features, and $o_{i}^{\left(c\right)}$ denotes the one-hot encoded categorical representation. This representation is suitable for tree-based and related tabular classifiers, whose learning mechanisms operate directly on explicit feature dimensions.

The transformer-oriented branch requires a different representational form. Numerical variables and engineered continuous features are preserved as scalar-valued inputs, while categorical variables are retained as discrete category indices for embedding-based projection. Let $c_{\mathrm{ij}}$ denote the category index of the j-th categorical attribute of sample i. Its embedding is defined as

$$e_{\mathrm{ij}}^{\left(c\right)}\;=\;W_{j}^{\left(c\right)}\;\mathrm{onehot}\;\left(c_{\mathrm{ij}}\right)$$

where $W_{j}^{\left(c\right)}$ is the learnable embedding matrix associated with the j-th categorical field. For a numerical variable $x_{\mathrm{ij}}$, the corresponding token representation is obtained through a linear projection,

$$e_{\mathrm{ij}}^{\left(n\right)}\;=\;W_{j}^{\left(n\right)}x_{\mathrm{ij}}\;+\;b_{j}^{\left(n\right)}$$

where $W_{j}^{\left(n\right)}$ and $b_{j}^{\left(n\right)}$ are learnable projection parameters. In this way, both continuous and categorical variables are mapped into a common latent space, enabling subsequent attention-based interaction modeling.

The resulting tabular input to the transformer can therefore be viewed as a sequence of feature tokens,

$$E_{i}\;=\;\left[e_{i1},e_{i2},\;\ldots ,\;e_{\mathrm{im}}\right]$$

where each token corresponds to one feature field or engineered attribute. This representation preserves field-level semantics while allowing nonlinear dependency learning across heterogeneous borrower characteristics. The role of representation design here is not merely technical compatibility; it also determines whether the model can meaningfully exploit the mixed-type structure of credit data.

#### 3.3.3. Preliminary Feature Analysis

Before formal model training, a preliminary feature analysis is conducted to examine the predictive relevance and statistical behavior of the available variables. This step serves two purposes. One is to provide an empirical basis for the subsequent model design; the other is to assess whether the original and engineered variables contain complementary information for credit prediction.

Feature relevance is first examined through mutual dependence and tree-based importance estimation. For a feature $x_{j}$ and the target variable y, the mutual information can be written as

$$I\left(x_{j};y\right)\;=\;\sum_{x_{j}}\;\sum_{y}\;p\left(x_{j},y\right)\log\frac{p\left(x_{j},y\right)}{p\left(x_{j}\right)p(y)}$$

which measures the statistical dependence between the predictor and the target label. A larger value indicates that the feature carries greater information about the class outcome. Since mutual information does not assume linear dependence, it is suitable for preliminary screening in credit risk data where nonlinear effects are common.

A complementary view is obtained from tree-based feature importance. Let $\Delta I_{j,t}$ denote the impurity reduction contributed by feature $x_{j}$ at split node t in an ensemble model. The cumulative importance score of feature $x_{j}$ can be expressed as

$$S_{j}^{\left(\mathrm{RF}\right)}\;=\;\sum_{t\in l_{j}}\;\Delta I_{j,t}$$

where $T_{j}$ denotes the set of nodes in which $x_{j}$ is used for splitting. This measure reflects the contribution of the feature to partition-based discrimination over the full ensemble. By combining information-theoretic dependence with tree-based relevance, the analysis provides a more stable view of which borrower attributes and engineered indicators are likely to play important roles in the classification task.

**Figure 4:**
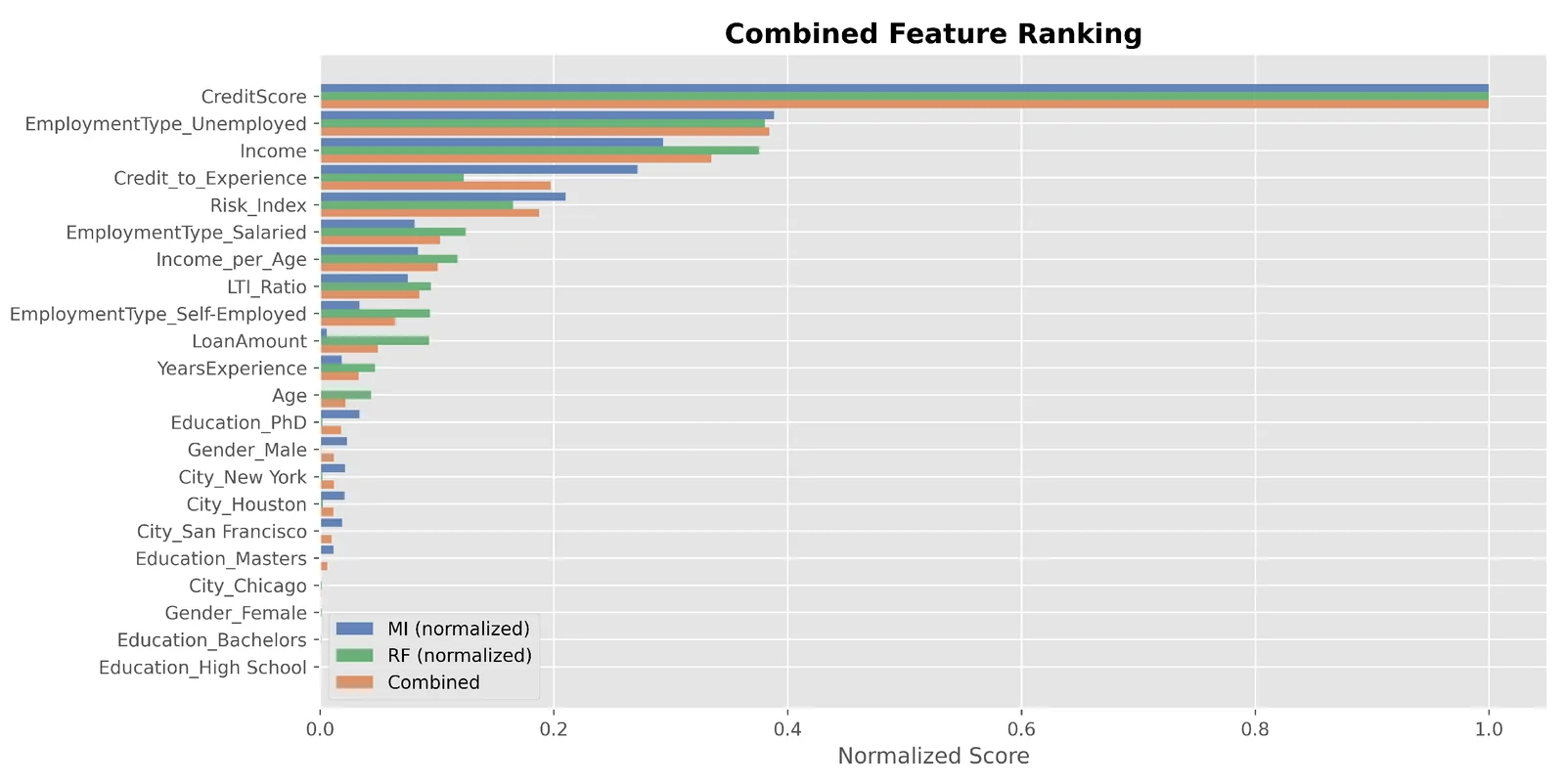
Combined feature ranking based on mutual information and Random Forest importance.

Figure 4 shows that CreditScore is the dominant predictor by a substantial margin, followed by EmploymentType_Unemployed and Income. Several engineered variables, including Credit_to_Experience, Risk_Index, Income_per_Age, and LTI_Ratio, also appear among the upper-ranked features, indicating that the feature construction stage adds meaningful predictive information beyond the original variable set. By contrast, several demographic and location-related indicators occupy the lower end of the ranking, suggesting weaker marginal contribution to the target outcome. This result provides direct empirical support for the subsequent model design and also anticipates the later explainability results, where the same variables emerge as principal drivers of the final predictions.

In addition to importance analysis, the feature space is examined from the perspective of redundancy and distributional structure. Correlation inspection among numerical variables and derived ratios is useful for identifying whether some engineered features introduce complementary predictive information or merely restate existing patterns in transformed form. This is particularly important in credit modeling, where overly aggressive feature construction may increase apparent complexity without improving actual representation quality. The preliminary analysis in this study is therefore used not as a rigid feature elimination stage, but as a diagnostic step that informs the subsequent design of the tabular learning framework and supports the interpretive analysis reported later in the paper.

### 3.4. Proposed Cross-Attentional Tabular Transformer

#### 3.4.1. Input Embedding and Cross-Attention Design

The proposed model is designed for heterogeneous credit data composed of continuous numerical attributes and discrete categorical attributes. Since these two variable types differ in representation form and statistical semantics, they are not processed identically at the input stage. The numerical branch preserves the continuous structure of scalar-valued borrower features, whereas the categorical branch maps discrete field values into dense embedding vectors. This separation enables the model to maintain field-level semantics while learning interactions in a shared latent space.

Let the numerical feature set of applicant i be denoted by

$$x_{i}^{\left(n\right)}\;=\;\left[x_{i1}^{\left(n\right)},x_{i2}^{\left(n\right)},\;\ldots ,\;x_{id_{n}}^{\left(n\right)}\right]$$

and let the categorical feature set be denoted by

$$x_{i}^{\left(c\right)}\;=\;\left[x_{i1}^{\left(c\right)},x_{i2}^{\left(c\right)},\;\ldots ,\;x_{id_{c}}^{\left(c\right)}\right]$$

Each numerical scalar is projected into a d-dimensional embedding space through an independent linear mapping,

$$e_{\mathrm{ij}}^{\left(n\right)}\;=\;W_{j}^{\left(n\right)}x_{\mathrm{ij}}^{\left(n\right)}\;+\;b_{j}^{\left(n\right)}$$

where $W_{j}^{\left(n\right)}\in R^{d}$ and $b_{j}^{\left(n\right)}\in R^{d}$ are learnable parameters associated with the j-th numerical field. This operation converts each continuous variable into a token-level representation while preserving its field identity.

For the categorical branch, each discrete field value is first mapped to an integer index and then embedded through a trainable lookup table. For the j-th categorical feature of sample i, the embedding is written as

$$e_{\mathrm{ij}}^{\left(c\right)}\;=\;W_{j}^{\left(c\right)}\left[x_{\mathrm{ij}}^{\left(c\right)}\right]$$

where $W_{j}^{\left(c\right)}\in R^{K_{j}\times d}$ denotes the embedding matrix of the j-th categorical field and $K_{j}$ is the number of valid categories in that field. In this way, numerical and categorical variables are transformed into tokens of the same latent dimension, allowing them to participate in a unified attention-based representation process.

After tokenization, the numerical and categorical embeddings are organized into two feature groups,

$$E_{i}^{\left(n\right)}\;=\;{\left[e_{i1}^{\left(n\right)},e_{i2}^{\left(n\right)},\;\ldots ,\;e_{id_{n}}^{\left(n\right)}\right]}^{⊤},\;E_{i}^{\left(c\right)}\;=\;{\left[e_{i1}^{\left(c\right)},e_{i2}^{\left(c\right)},\;\ldots ,\;e_{id_{c}}^{\left(c\right)}\right]}^{⊤}$$

Rather than directly merging all tokens and relying only on standard self-attention, the proposed model introduces a cross-attention mechanism to capture dependency exchange between heterogeneous feature groups. The motivation is that, in credit assessment, the predictive effect of a financial variable is often conditioned by demographic or employment-related categories, while categorical risk patterns are likewise shaped by quantitative financial exposure. A mechanism that explicitly allows one feature group to attend to the other is therefore better aligned with the structure of the problem than a purely undifferentiated token interaction scheme.

Given a query matrix Q, key matrix K, and value matrix V, the scaled dot-product attention is defined as

$$\mathrm{Attn}(Q,K,V)=\mathrm{softmax}\left(\frac{QK^{⊤}}{\sqrt{d}}\right)V$$

In the proposed cross-attentional design, one branch is used to generate query representations, while the other supplies the corresponding keys and values. When the numerical branch attends to the categorical branch, the cross-attention output is written as

$$H_{i}^{\left(n+\text{-}c\right)}\;=\;\mathrm{Attn}\left(E_{i}^{\left(n\right)}W_{Q}^{\left(n\right)},E_{i}^{\left(c\right)}W_{K}^{\left(c\right)},E_{i}^{\left(c\right)}W_{V}^{\left(c\right)}\right)$$

Similarly, when the categorical branch attends to the numerical branch, the reverse interaction is given by

$$H_{i}^{\left(c<\text{-}n\right)}\;=\;\mathrm{Attn}\left(E_{i}^{\left(c\right)}W_{Q}^{\left(c\right)},E_{i}^{\left(n\right)}W_{K}^{\left(n\right)},E_{i}^{\left(n\right)}W_{V}^{\left(n\right)}\right)$$

where $W_{Q}^{\left(\cdot \right)},W_{K}^{\left(\cdot \right)}$, and $W_{V}^{\left(\cdot \right)}$ are learnable projection matrices. These bidirectional cross-attention operations allow the model to learn how continuous financial quantities and discrete borrower profiles condition one another. The fused token representation is then obtained by combining the original embeddings with the cross-attended outputs,

$${\tilde{D}}_{i}\;=\;\mathrm{Concat}\left(E_{i}^{\left(n\right)}\;+\;H_{i}^{(n\leq \text{-}c)},E_{i}^{\left(c\right)}\;+\;H_{i}^{(c\leq \text{-}n)}\right)$$

This representation serves as the input to the subsequent transformer encoder.

#### 3.4.2. Transformer Encoder and Classification Head

The fused token matrix ${\tilde{D}}_{i}$ is processed by a transformer encoder to capture higher-order dependencies across all feature fields. To obtain a sample-level representation for binary classification, a learnable classification token $e_{\mathrm{cls}\;}$ is prepended to the token sequence. The resulting encoder input is

$$z_{i}^{\left(0\right)}\;=\;\mathrm{Concat}\left(e_{\mathrm{cls}},{\tilde{D}}_{i}\right)$$

This design allows the classification token to aggregate information from the full feature set through attention propagation across encoder layers.

Each encoder block consists of a multi-head self-attention sublayer followed by a position-wise feed-forward network. Given the input $Z_{i}^{\left(l\text{-}1\right)}$ to the l-th encoder layer, the attention sublayer computes

$$A_{i}^{\left(l\right)}\;=\;\mathrm{MHA}\left(Z_{i}^{\left(l\text{-}1\right)}\right)$$

where MHA denotes multi-head self-attention. The output of the attention sublayer is combined with the input through a residual connection and layer normalization,

$$U_{i}^{\left(l\right)}\;=\;\mathrm{LayerNorm}\left(Z_{i}^{\left(l\text{-}1\right)}\;+\;A_{i}^{\left(l\right)}\right)$$

The normalized representation is then passed through a feed-forward network,

$$F_{i}^{\left(l\right)}\;=\;\mathrm{FFN}\left(U_{i}^{\left(l\right)}\right)$$

followed by a second residual connection and normalization step,

$$Z_{i}^{\left(l\right)}\;=\;\mathrm{LayerNorm}\;\left(U_{i}^{\left(l\right)}\;+\;F_{i}^{\left(l\right)}\right)$$

This structure preserves training stability while allowing successive nonlinear transformations of the feature interactions learned through attention.

The feed-forward component is implemented as a two-layer nonlinear mapping,

$$\mathrm{FFN}(u)=W_{2}\sigma \left(W_{1}u\;+\;b_{1}\right)\;+\;b_{2}$$

where σ(·) denotes the activation function. In the present model, a smooth nonlinear activation is adopted to improve representation continuity for tabular inputs. Dropout is further applied within the encoder to reduce overfitting and improve generalization under limited sample size.

After the final encoder layer, the hidden state associated with the classification token is extracted as the sample-level representation,

$$h_{i}\;=\;Z_{i,\mathrm{cls}}^{\left(L\right)}$$

where L denotes the number of encoder layers. The final prediction is produced by a multilayer classification head,

$${\hat{p}}_{i}\;=\;\mathrm{sigmoid}\left(W_{o}\phi \left(W_{h}h_{i}\;+\;b_{h}\right)\;+\;b_{o}\right)$$

where ϕ(·) is a nonlinear activation function and ${\hat{p}}_{i}\in (0,\;1)$ denotes the predicted probability of the positive class. The classification head transforms the global representation learned by the encoder into a scalar risk score suitable for binary credit assessment.

This architecture differs from conventional tabular transformers in that the interaction between numerical and categorical variables is explicitly structured before global self-attention is applied. The encoder therefore operates on a representation that already incorporates directed cross-type dependency exchange, which is particularly relevant in credit data where financial and demographic attributes often exert conditional effects on one another.

#### 3.4.3. Imbalance-Aware Optimization

Credit risk assessment is inherently sensitive to class imbalance because the minority class often corresponds to the outcome of greater practical interest. Under such conditions, optimization based solely on standard binary cross-entropy may lead the model to favor the majority class, thereby producing inflated overall accuracy but insufficient sensitivity to minority-class cases. Since the practical objective of credit risk modeling is not merely overall correctness but effective recognition of adverse outcomes, the training objective must be adjusted accordingly.

For a sample with label $y_{i}\in \{0,1\}$ and predicted probability ${\hat{p}}_{i}$, the standard binary cross-entropy loss is

$$L_{\mathrm{BCE}}\;=\;-\left[y_{i}\log\left({\hat{p}}_{i}\right)\;+\;\left(1-y_{i}\right)\log\left(1-{\hat{p}}_{i}\right)\right]$$

This formulation assigns equal structural importance to all samples. When the majority class dominates the training data, the loss gradient is also dominated by easy-to-classify majority instances, which may weaken the model’s attention to rare but operationally important minority cases.

To alleviate this bias, class-weighted learning is introduced by assigning different coefficients to the two classes,

$$L_{\mathrm{WBCE}}\;=\;-\left[\alpha_{1}y_{i}\log\left({\hat{p}}_{i}\right)\;+\;\alpha_{0}\left(1-y_{i}\right)\log\left(1-{\hat{p}}_{i}\right)\right]$$

where $\alpha_{1}$ and $\alpha_{0}$ are the class weights associated with the positive and negative classes, respectively. This weighting scheme amplifies the contribution of the minority class during optimization and improves the alignment between training dynamics and minority-class-sensitive classification objectives.

To further emphasize difficult samples, the proposed framework adopts focal-style modulation. Let

$$p_{t,i}\;=\;\left\{\begin{array}{cc} {\hat{p}}_{i}, & y_{i}\;=\;1\\ 1-{\hat{p}}_{i}, & y_{i}\;=\;0 \end{array}\right.\;$$

The focal loss is then written as

$$L_{\mathrm{focal}\;}\;=\;-\alpha_{t,i}{\left(1-p_{t,i}\right)}^{\gamma }\log\left(p_{t,i}\right)$$

where $\alpha_{t,i}$ denotes the class-dependent balancing factor and γ≥0 is the focusing parameter. When a sample is already classified with high confidence, $p_{t,i}$ approaches 1 and the modulating factor ${\left(1-p_{t,i}\right)}^{\gamma }$ suppresses its loss contribution. Conversely, hard samples with low $p_{t,i}$ retain larger loss values and therefore receive stronger optimization emphasis.

This mechanism is particularly appropriate for imbalanced credit data. Majority-class samples that are easily separated do not dominate parameter updates, while difficult minority cases remain influential throughout training. As a result, the model is encouraged to allocate more representational capacity to the decision boundary regions most relevant to minority-class identification. In the proposed framework, the imbalance-aware objective is used to improve minority-class sensitivity without altering the fundamental probabilistic interpretation of the classifier output. This allows the final model to preserve a standard binary prediction interface while exhibiting stronger robustness under skewed class distributions.

## 4. Results

### 4.1. Experimental Setup

The experiments were conducted on the processed credit risk dataset derived from 5000 loan application records. After data cleaning and preprocessing, the samples were divided into training and testing subsets using an 80%/20% split, yielding 4000 training samples and 1000 testing samples. The evaluation was performed under an imbalanced binary classification setting, in which the negative class remained dominant throughout both subsets. The main comparative analysis includes Random Forest and Gradient Boosting as established structured-data baselines and the proposed Cross-Attentional Tabular Transformer as an attention-based heterogeneous representation model. Section 4.2 additionally reports an extended, five-split robustness comparison against XGBoost, LightGBM, CatBoost, and calibrated logistic regression on the primary dataset. The experimental comparison is restricted to this baseline set; deep tabular architectures such as TabNet, FT-Transformer, SAINT, and TabPFN-style small-data methods fall outside its scope.

Model performance was assessed from both threshold-dependent and threshold-independent perspectives. Accuracy, precision, recall, and F1-score were used to evaluate classification quality at the decision level, while ROC-AUC and average precision were employed to examine ranking ability and minority-class discrimination under imbalanced conditions. In addition to predictive performance, training efficiency and class-specific behavior were also analyzed in order to provide a more complete comparison across model families. For interpretability, SHAP was applied after model fitting to identify globally important predictors and to characterize local feature contributions, thereby linking quantitative performance evaluation with transparent credit decision analysis.

### 4.2. Baseline Performance Comparison

The comparative results indicate that all three models achieved strong predictive performance on the credit risk assessment task, but clear differences remained in both classification quality and computational efficiency. Gradient Boosting delivered the best overall fixed-threshold performance, with an accuracy of 0.9640, an F1-score of 0.9189, a precision of 0.9533, and an average precision of 0.9106. Random Forest followed closely, attaining an accuracy of 0.9630, an F1-score of 0.9169, and the highest ROC-AUC of 0.9407, which suggests slightly stronger threshold-independent ranking ability. The proposed transformer-based model remained competitive, reaching an accuracy of 0.9530 and an F1-score of 0.8949, but it did not surpass the two ensemble baselines under the current data scale. A substantial difference also appeared in training cost.

**Figure 5:**
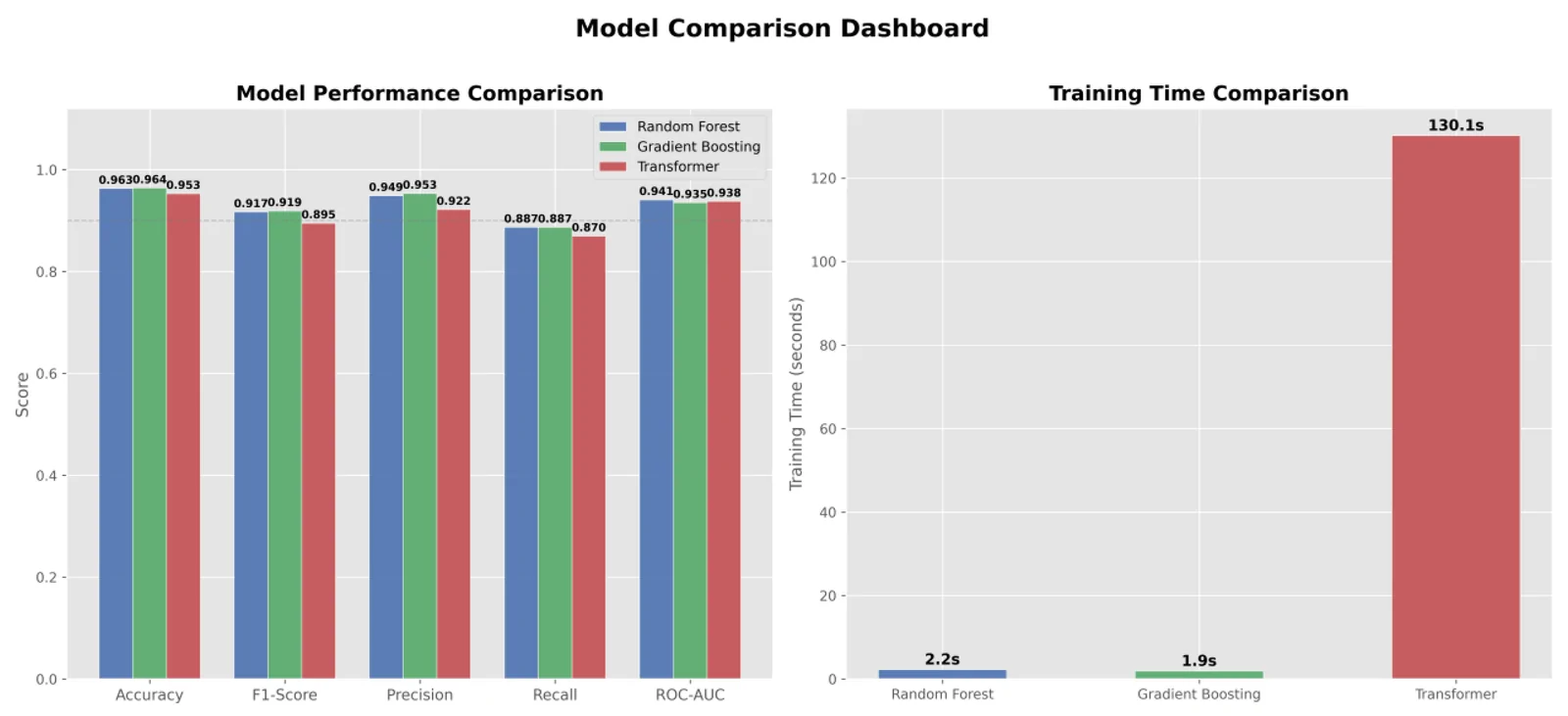
Overall performance and training-time comparison of Random Forest, Gradient Boosting, and the proposed transformer-based model.

Figure 5 provides a direct comparison of the three models in terms of predictive performance and computational cost. Gradient Boosting achieved the best fixed-threshold classification results, with the highest accuracy, F1-score, precision, and average precision, indicating the strongest overall balance between classification correctness and minority-class recognition. Random Forest showed nearly identical predictive quality and retained the highest ROC-AUC, suggesting slightly stronger threshold-independent ranking ability. Although the transformer-based model remained competitive across all metrics, it did not exceed the ensemble baselines under the current data scale. The training time comparison further highlights a substantial efficiency gap: while the two tree-based models completed training within only a few seconds, the transformer required more than two orders of magnitude longer training time.

While Random Forest and Gradient Boosting completed training within only a few seconds, the transformer required approximately 130 s, indicating that its additional representational flexibility did not translate into a superior performance–efficiency trade-off in the present static tabular setting. Section 5.2 further discusses this cost–performance comparison.

To assess whether this ranking is specific to the two classical ensemble methods considered above, and whether it is sensitive to the particular fixed split used in the main comparison, six low-cost tabular baselines, Random Forest, Gradient Boosting, three further Gradient-Boosting variants (XGBoost, LightGBM, CatBoost), and a calibrated logistic regression model, were retrained and re-evaluated across five independent stratified 80%/20% splits of the primary dataset, using the same traditional feature representation as the main comparison.

Averaged across five splits, all five tree-based and Gradient-Boosting methods (Random Forest, Gradient Boosting, XGBoost, LightGBM, and CatBoost) exceed the proposed transformer’s single-split accuracy, precision, recall, F1-score, ROC-AUC, and average precision, while training in well under one second on average compared with approximately 130 s for the transformer. Calibrated logistic regression, by contrast, falls below the transformer on every performance metric, indicating that the transformer’s representational capacity provides a meaningful advantage over a simple linear baseline even though it does not close the gap with tree-based ensemble methods. The tree-based and Gradient-Boosting results are five-split averages, whereas the transformer result reflects a single fixed split; consequently, their cross-protocol comparison is descriptive rather than a controlled statistical test. Table 6 summarizes the repeated-split results.

A more detailed inspection of the classification structure shows that the primary performance gap emerged in the minority Approved class rather than in the dominant Not Approved class.

**Figure 6:**
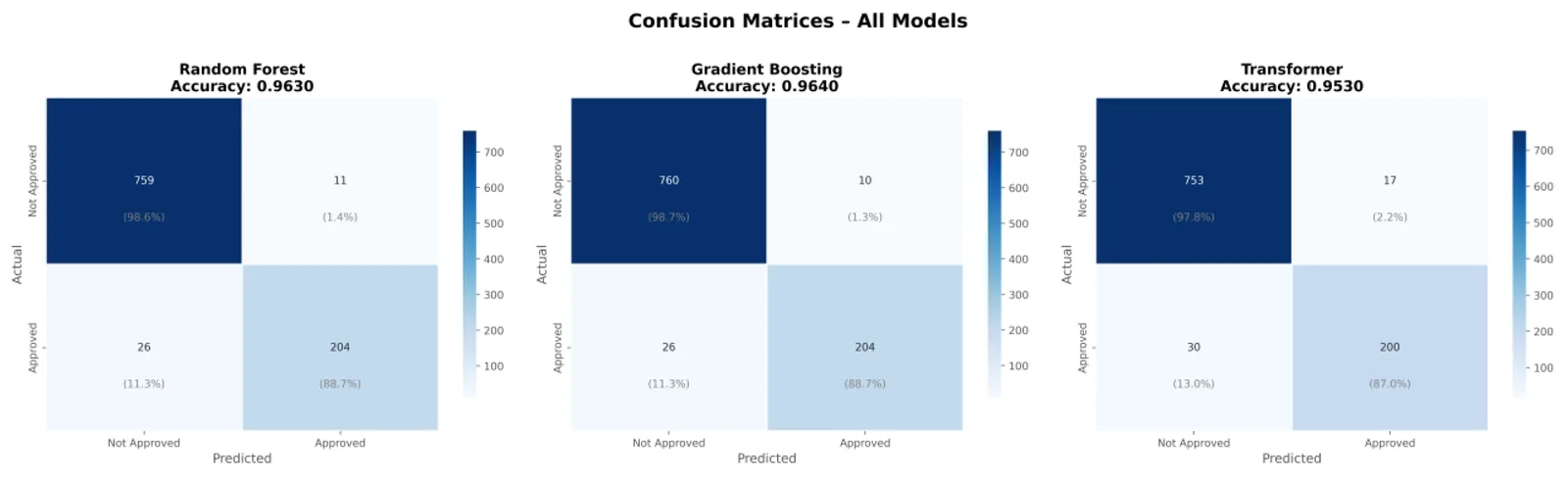
Confusion matrices of the three models on the test set.

Figure 6 shows that all three models achieved very strong recognition of the dominant Not Approved class, as reflected by the high true-negative counts and the low number of false approvals. The main performance gap is observed in the Approved class, where the two ensemble models preserved a more favorable balance between false negatives and true positives. Gradient Boosting and Random Forest produced nearly identical confusion structures, whereas the transformer introduced slightly more false negatives and false positives. This pattern confirms that the difference among models is not primarily determined by majority-class discrimination, which is strong for all methods, but by the ability to maintain stable recognition of the minority Approved class under an imbalanced setting.

Both ensemble models maintained very strong recognition of the majority class while also preserving better balance on the minority class, as reflected by their higher Approved-class F1-score and recall.

**Figure 7:**
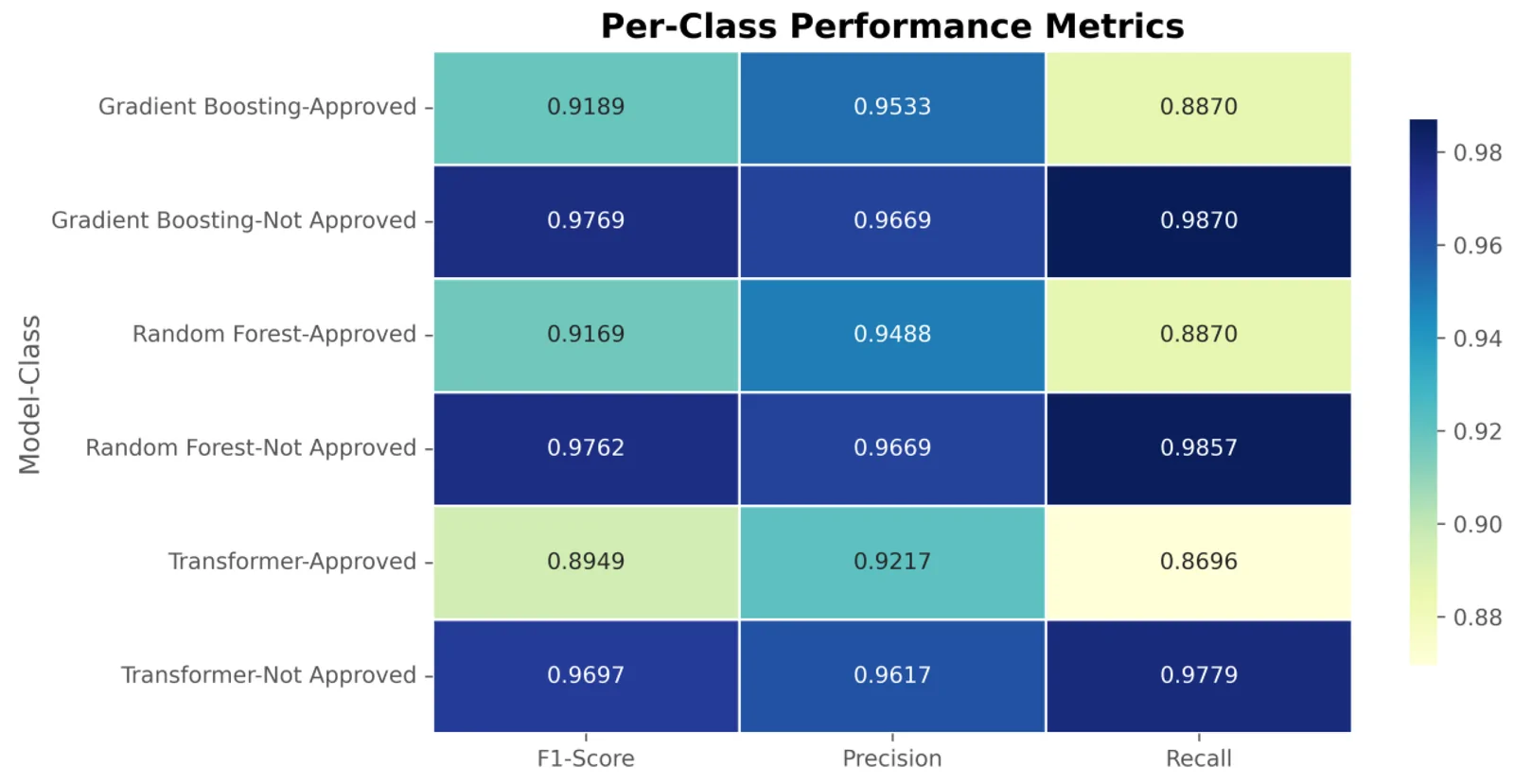
Per-class precision, recall, and F1-score of the three models.

Figure 7 further decomposes model performance at the class level and confirms that the most meaningful differences arise in the Approved class. Gradient Boosting obtained the highest F1-score and precision for Approved samples, while Random Forest remained extremely close and shared the same recall value. By contrast, the transformer showed lower values across all three Approved-class metrics, especially in recall, indicating reduced sensitivity to minority positive cases. For the Not Approved class, all models performed strongly and remained close to one another, with only marginal differences. This result demonstrates that model ranking in the present task is largely governed by the stability of minority-class prediction rather than majority-class correctness.

Gradient Boosting and Random Forest produced nearly identical true-positive counts, whereas the transformer introduced more false negatives and false positives, leading to a weaker balance between minority-class sensitivity and precision. This pattern is consistent with the precision–recall analysis, where Gradient Boosting achieved the highest area under the PR curve, followed closely by Random Forest, while the transformer remained slightly lower across much of the recall range.

**Figure 8:**
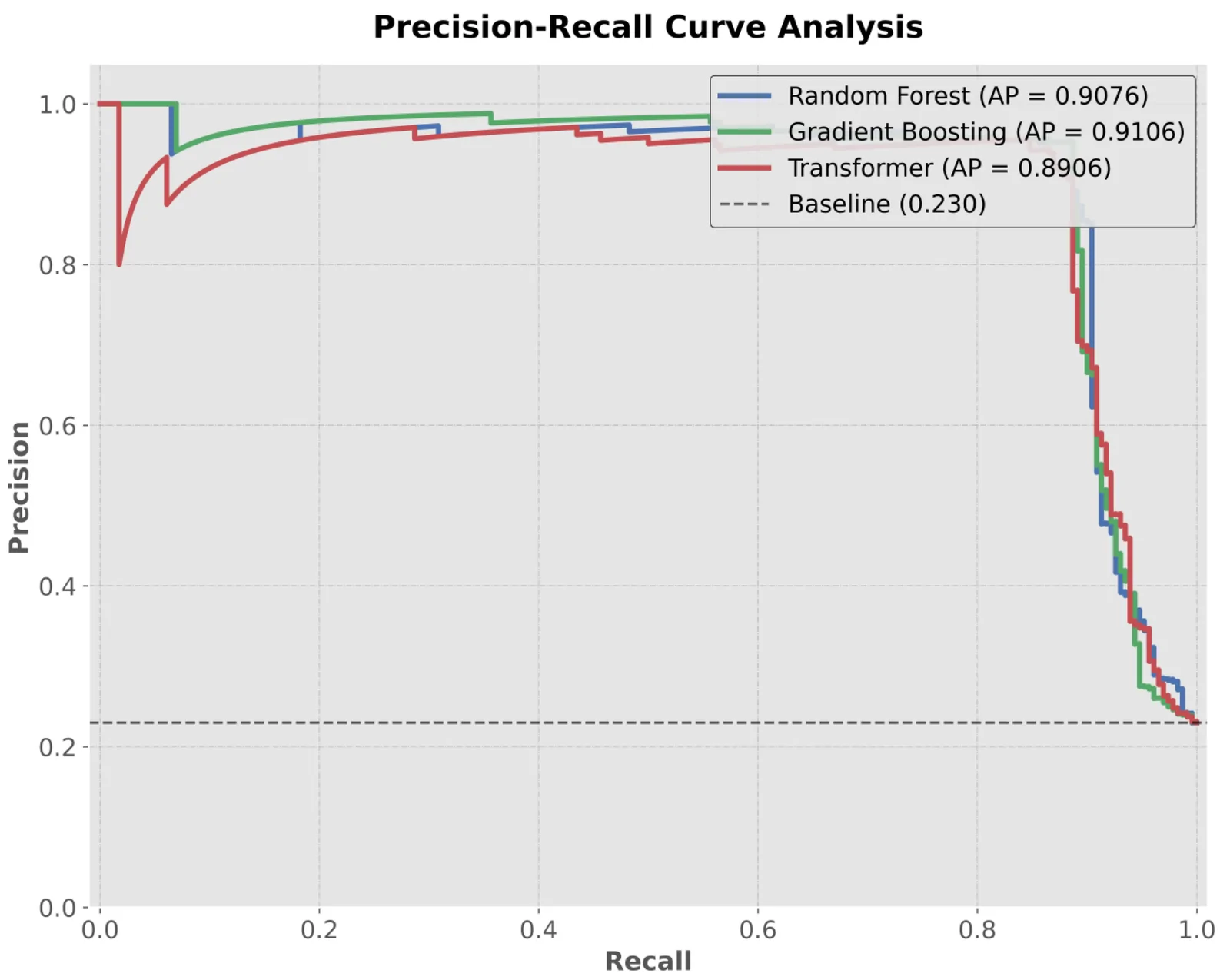
Precision–recall curves of the three models under imbalanced classification.

Figure 8 emphasizes model behavior from the perspective most relevant to imbalanced classification. All three methods performed far above the baseline corresponding to random positive prediction, confirming substantial predictive value for the Approved class. Nevertheless, the ranking among models remained clear. Gradient Boosting achieved the highest average precision, indicating the strongest overall precision–recall trade-off across thresholds. Random Forest followed closely and exhibited similarly strong minority-class discrimination. The transformer remained competitive but showed a slightly weaker curve over large portions of the recall axis, suggesting that higher recall was obtained at the cost of a faster reduction in precision. These results reinforce the conclusion that the ensemble models are better suited to preserving positive-class discrimination quality in the current credit risk setting.

The ROC analysis further refines this comparison by showing that Random Forest retained the strongest overall ranking ability across thresholds, even though Gradient Boosting was marginally superior under the default decision threshold.

**Figure 9:**
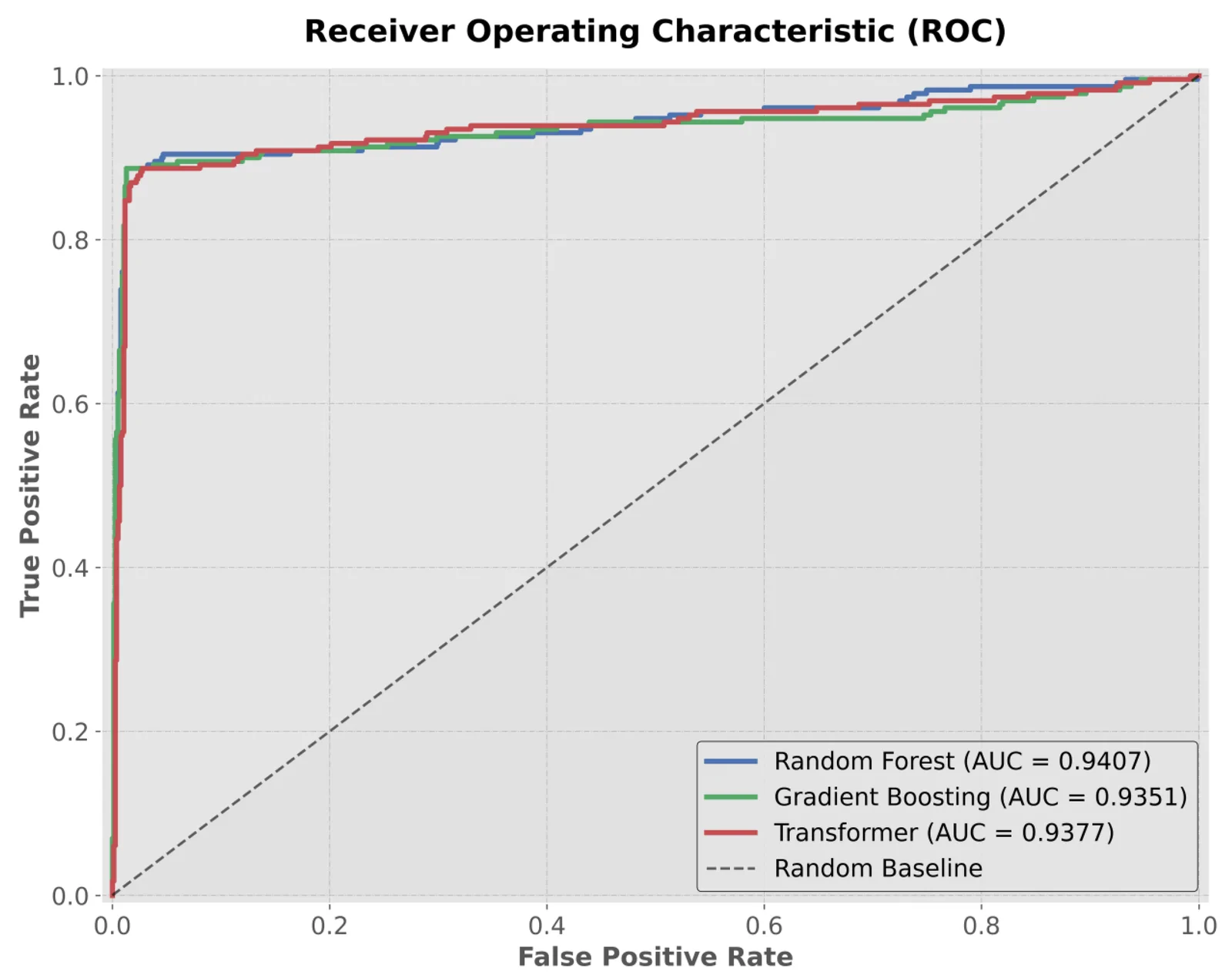
ROC curves of the three models on the credit risk test set.

Figure 9 complements the fixed-threshold and precision–recall results by examining threshold-independent ranking performance. The three ROC curves are all located far above the diagonal reference line, indicating strong discriminative ability across models. Among them, Random Forest achieved the highest AUC, albeit by a narrow margin, which suggests a slight advantage in overall probability ranking quality. Gradient Boosting remained extremely close, indicating that its superiority in fixed-threshold classification was not due to a universally dominant score distribution, but rather to a more favorable decision outcome under the adopted threshold. The transformer again showed competitive but not leading performance.

Taken together, these results indicate that ensemble tree models remain the most effective solutions for the present credit risk dataset, while the proposed transformer verifies the feasibility of attention-based tabular learning without yet exceeding the strongest conventional baselines.

### 4.3. Ablation Study on the Bidirectional Cross-Attention Mechanism

To validate the contribution of the proposed bidirectional cross-attention mechanism, an ablation experiment was conducted comparing the proposed model against a vanilla TabTransformer baseline. In the baseline variant, all numerical and categorical feature tokens were directly concatenated into a single sequence and processed by standard multi-head self-attention without any structural distinction between the two feature types, following the conventional TabTransformer design. In contrast, the proposed model performs bidirectional cross-attention between the numerical and categorical feature groups prior to global self-attention encoding, explicitly modeling dependency exchange between the two heterogeneous feature types. All other experimental settings were held constant across both variants, including data partitioning, feature preprocessing, embedding dimension, number of attention heads, number of encoder layers, optimizer, focal loss objective, dropout rate, and early-stopping strategy. Performance differences between the two variants can therefore be attributed solely to the attention mechanism design.

As shown in Table 7, the proposed model achieves consistent improvements across all evaluation metrics. The F1-score increases from 0.8865 to 0.9043, ROC-AUC from 0.9274 to 0.9386, and average precision from 0.8956 to 0.9128. The most substantial gain is observed in recall, which improves from 0.8618 to 0.8880. Improved recall is particularly meaningful under the approval-status label used in this study, because it indicates that the proposed mechanism reduces the number of Approved-class applicants incorrectly classified as Not Approved, an error category that carries direct consequences for applicants who would otherwise qualify.

The confusion matrices in Table 8 provide a more granular view of the improvement. The proposed model reduces false negatives from 35 to 28, indicating stronger identification of applicants who were actually Approved. Simultaneously, false positives are reduced from 21 to 19 and true positives increase from 215 to 222, reflecting a more balanced improvement across the minority class rather than a simple precision–recall trade-off. Under the approval-status label used in this study, false negatives correspond to applicants who were actually Approved but predicted as Not Approved, and their reduction carries greater practical importance than equivalent gains in majority-class accuracy.

Taken together, these results confirm that the bidirectional cross-attention mechanism provides a moderate but consistent improvement over a vanilla self-attention baseline on concatenated tokens. The gains are observed uniformly across threshold-dependent and threshold-independent metrics, supporting the conclusion that the explicit modeling of numerical–categorical interaction dependencies contributes meaningfully to the representational quality of the proposed framework. Table 7 and Table 8 report an independently retrained controlled ablation and therefore represent a different trained instance from the main baseline comparison in Section 4.2.

### 4.4. Ablation Study on Engineered Financial Ratio Features

To examine the contribution of the three engineered financial ratio features, a second ablation experiment was conducted. The proposed transformer was evaluated under two settings: one using only the original numerical and categorical variables (together with the income-per-age indicator described in Section 3.3.1, which is not toggled in this ablation), and the other additionally incorporating the loan-to-income ratio, the credit-to-experience ratio, and the risk index. These three features are designed to capture financially meaningful relationships that are not directly represented by the original variables. Specifically, the loan-to-income ratio reflects applicant repayment burden relative to income; the credit-to-experience ratio captures the relationship between credit quality and accumulated professional experience; and the risk index describes loan exposure relative to credit quality, with higher values corresponding to greater exposure relative to demonstrated creditworthiness. All other experimental settings, including data partitioning, feature preprocessing, model architecture, bidirectional cross-attention mechanism, loss function, optimizer, and early-stopping strategy, were held constant across both settings, so that performance differences can be attributed solely to the inclusion or exclusion of the ratio features.

As shown in Table 9, incorporating the ratio features produces consistent improvements across all evaluation metrics. The F1-score increases from 0.8840 to 0.9043, ROC-AUC from 0.9251 to 0.9386, and average precision from 0.8914 to 0.9128. The most notable gain is in recall, which improves from 0.8560 to 0.8880. The recall improvement is particularly relevant because it indicates that the ratio features assist the model in correctly identifying additional Approved-class applicants that are otherwise missed by the original feature representation alone.

The confusion matrices in Table 10 provide a more granular view of this effect. Removing the ratio features increases the number of false negatives from 28 to 36, indicating that a meaningful share of applicants who were actually Approved are no longer correctly identified when the engineered relational indicators are absent. The number of false positives also increases slightly from 19 to 20, and true positives decrease from 222 to 214. This pattern confirms that the ratio features contribute primarily to minority-class recognition rather than to majority-class classification, which is already strong in both settings.

These results indicate that the engineered financial ratio features provide complementary risk information that the model does not fully recover from the original numerical and categorical variables alone. The performance improvement of the proposed framework therefore reflects the combined effect of transformer-based heterogeneous representation learning and financially meaningful feature construction. The cross-attentional architecture and domain-informed ratio features jointly contribute to predictive quality. Table 9 and Table 10 report an independently retrained feature-ablation instance distinct from the main trained instance in Section 4.2.

### 4.5. Sensitivity and Training Behavior Analysis

Although the proposed transformer-based model did not outperform the strongest ensemble baselines in overall classification accuracy, its training dynamics remained stable throughout the optimization process. The loss curves show that both training loss and testing loss decreased rapidly during the early epochs and then converged within a relatively narrow range, indicating that the model was able to capture the principal data structure without exhibiting unstable oscillation or late-stage divergence. A similar pattern is observed in the accuracy curves, where training and testing accuracy increased quickly and remained close to one another after the initial learning phase.

**Figure 10:**
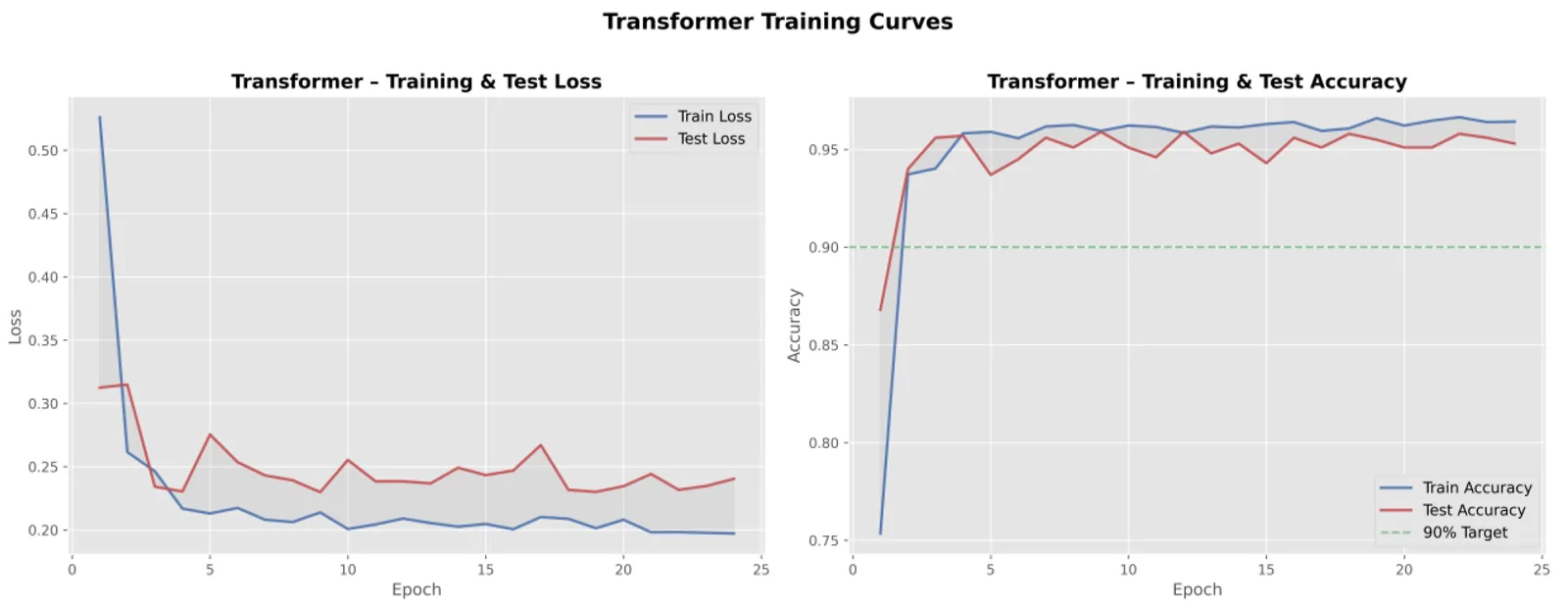
Training and validation loss and accuracy curves of the proposed transformer-based model.

Figure 10 shows that the proposed transformer converged in a stable and controlled manner during training. Both loss curves declined sharply in the early stage and then gradually flattened, indicating that the main predictive structure of the data was captured within a limited number of epochs. The testing loss remained slightly higher than the training loss, but the gap was small and did not widen over time, which suggests that overfitting was effectively constrained. The accuracy curves exhibit a similar tendency, with both training and testing accuracy reaching stable levels after early optimization. These observations confirm that the transformer was properly optimized under the adopted training strategy.

The limited gap between the two curves suggests that the final performance of the transformer was not constrained by severe overfitting, but rather by the representational and statistical characteristics of the current task setting.

From a sensitivity perspective, the observed convergence behavior indicates that the proposed architecture is trainable and numerically well behaved under the adopted optimization configuration. The model reached a stable performance plateau after a relatively small number of epochs, which implies that the current dataset does not require prolonged training to extract the dominant predictive signals. At the same time, the fact that the converged testing performance remained slightly below that of Gradient Boosting and Random Forest suggests that the main limitation of the transformer in this study is not optimization failure, but the insufficient conversion of its representational flexibility into measurable predictive gains on a moderate-scale tabular dataset. This result is important for methodological interpretation, as it indicates that the value of the proposed model lies more in its extensibility, interaction modeling capacity, and compatibility with richer feature representations than in immediate superiority over mature tree-based methods under the present experimental conditions.

### 4.6. Explainability Results

The SHAP analysis reveals that the prediction mechanism of the proposed framework is dominated by a small group of financially meaningful variables rather than by diffuse contributions from many weak predictors. Across the global explanation results, Credit Score consistently appears as the most influential feature, followed by Employment Type Unemployed and Income.

**Figure 11:**
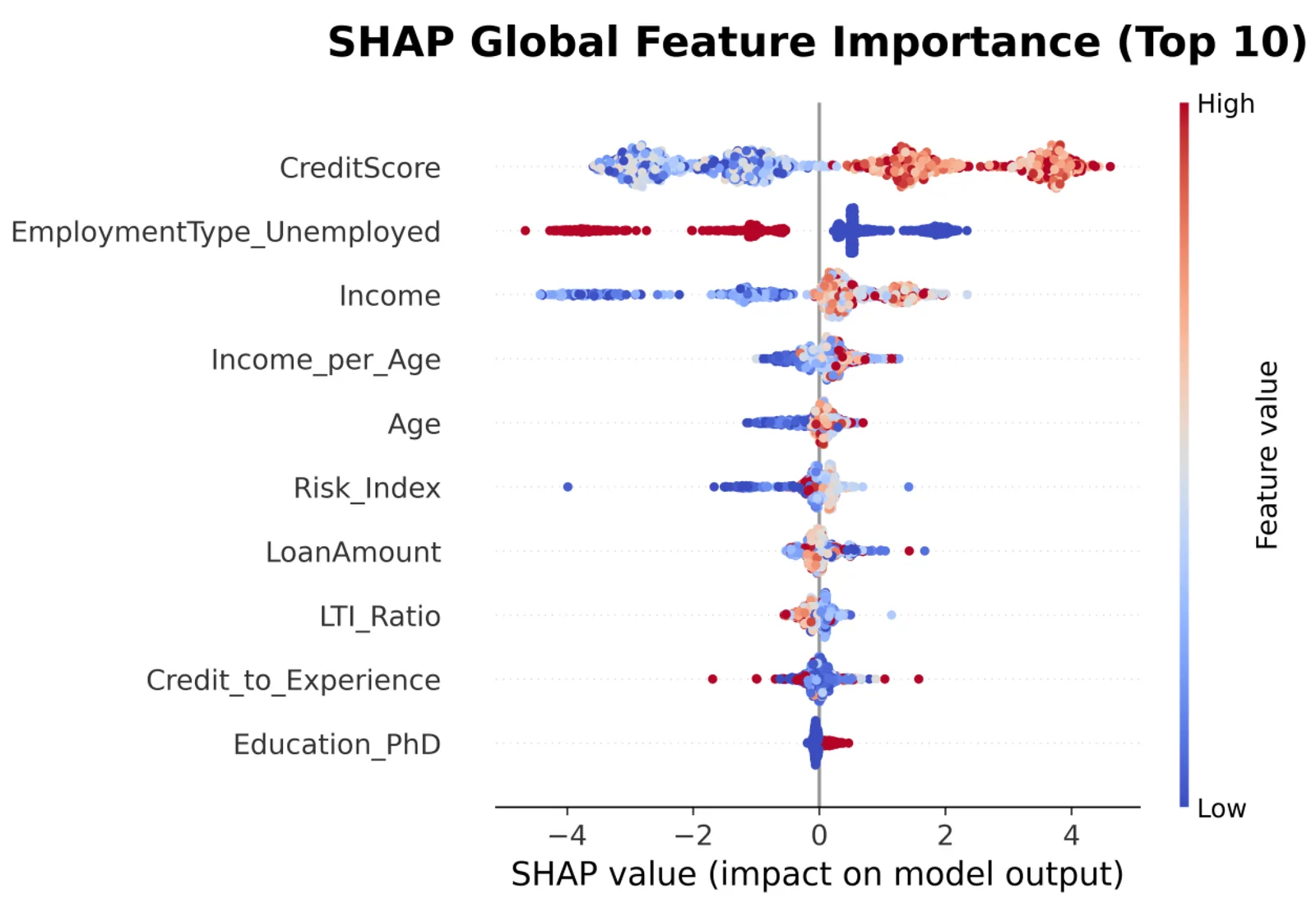
SHAP summary plot showing the global importance and directional effects of the most influential features.

Figure 11 summarizes the global contribution pattern of the most important predictors and shows that the model relies primarily on a compact set of financially meaningful variables. CreditScore occupies the dominant position, with high values contributing positively to the approval prediction and low values exerting a clear negative effect. EmploymentType_Unemployed exhibits a strongly negative directional pattern, indicating that unemployment status consistently suppresses the predicted approval probability. Income also shows a predominantly positive contribution, although its spread is more gradual than that of CreditScore. The directional SHAP distribution therefore confirms that the proposed model captures decision rules that are both interpretable and aligned with domain expectations in credit risk assessment.

This ranking is highly consistent with the earlier statistical analysis and feature relevance results, indicating that the model concentrates its decision logic on variables with clear financial interpretation. The global SHAP distributions further show that higher credit scores and higher income values generally contribute positively to the predicted approval outcome, whereas unemployment status produces a pronounced negative contribution.

**Figure 12:**
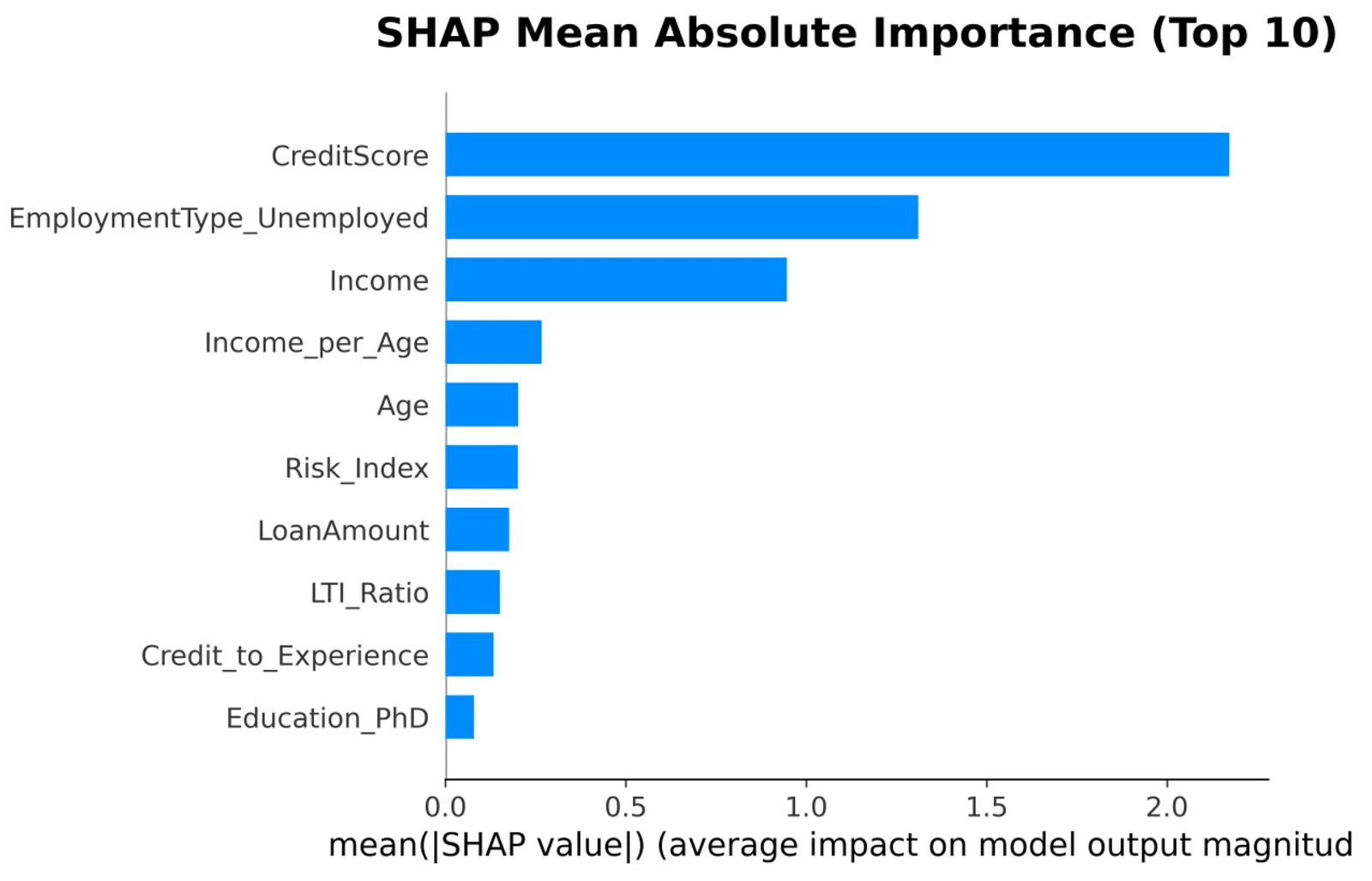
Mean absolute SHAP values of the top-ranked features.

Figure 12 provides a more compact ranking of feature importance based on the mean absolute SHAP value and makes the hierarchy among predictors clearer. CreditScore shows the largest average contribution by a substantial margin, indicating that it is the principal driver of the model output at the portfolio level. EmploymentType_Unemployed and Income form the second tier of influence, while the remaining variables contribute mainly as supporting adjustments to the main risk structure. This concentration of importance implies that the model does not distribute decision weight arbitrarily across many weak predictors, but instead bases its judgment on a small number of dominant variables with clear business interpretation.

This pattern suggests that the model has captured a decision structure aligned with the basic logic of credit evaluation, in which credit quality, repayment capacity, and employment stability jointly shape the predicted risk level.

The dominant role of CreditScore warrants further contextual interpretation. Standardized credit scores, such as FICO in the United States, are designed precisely to aggregate a range of borrower repayment signals into a single scalar indicator, so their strong marginal predictive value in a lending context is not surprising. However, the substantial residual contributions of EmploymentType_Unemployed and Income in the present SHAP analysis indicate that credit score alone does not fully characterize loan outcome risk. Employment instability and income-relative loan exposure represent risk dimensions that are not directly encoded in a standard credit score calculation and that remain independently informative in the model’s decision structure. This observation reflects a practical distinction between using a credit score as a screening threshold and using a multi-feature model to identify contextual risk factors that the score does not capture. The former is operationally simpler and highly interpretable, but it leaves a measurable share of the predictive signal uncaptured, particularly in borderline applicant profiles where no single indicator provides confident discrimination. The value of the proposed multi-feature framework lies precisely in this residual explanatory power and in the interaction effects between credit quality, income, and employment status that it learns to represent.

The local dependence analysis provides additional evidence that the model is not merely aggregating independent feature effects, but is learning conditional interactions among key borrower attributes. The dependence plot of CreditScore shows a clear transition from negative to positive SHAP values as the score increases, indicating a strong monotonic effect with an evident threshold-like structure.

**Figure 13:**
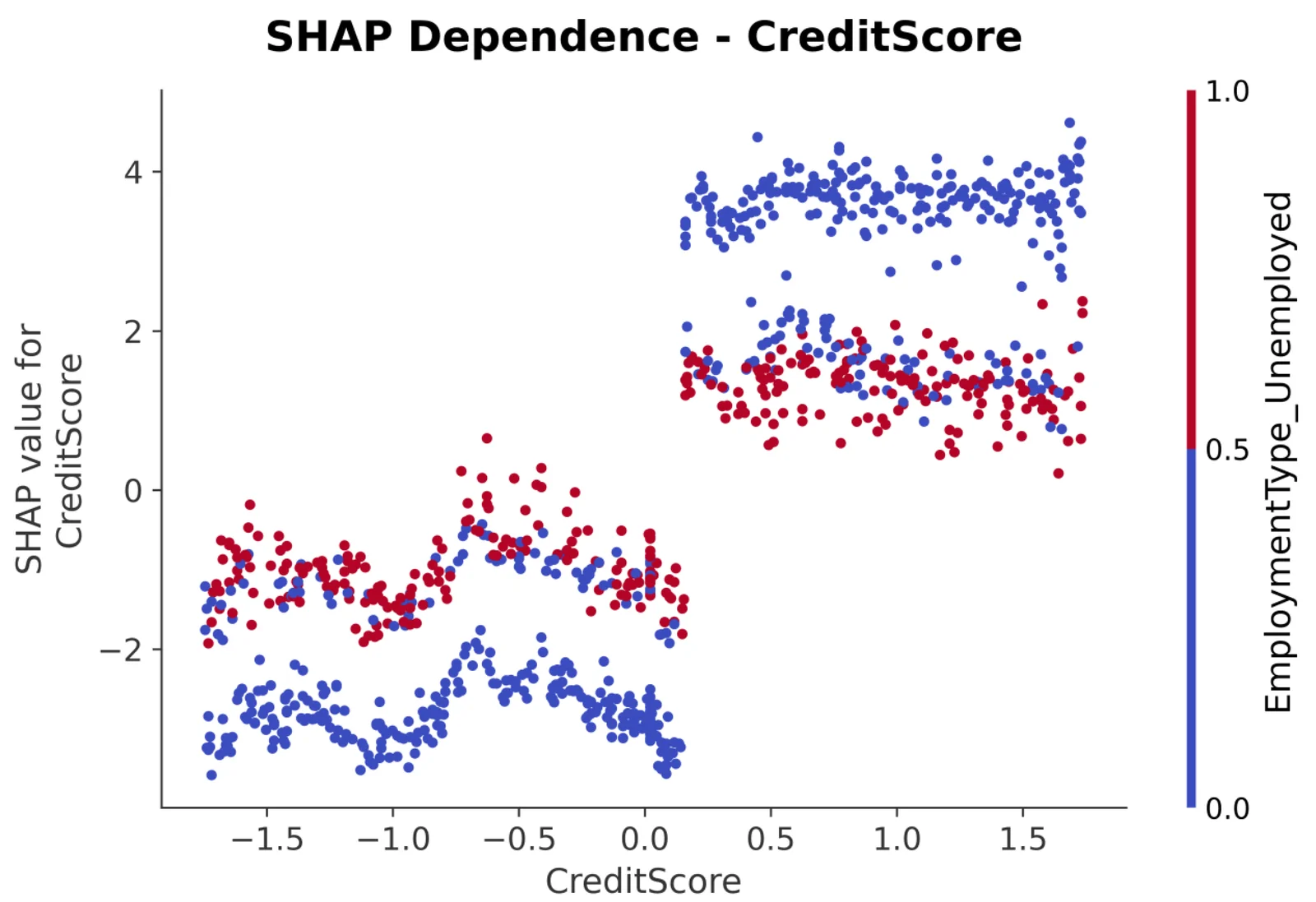
SHAP dependence plot of CreditScore with interaction coloring by EmploymentType_Unemployed.

Figure 13 shows that the contribution of CreditScore is not merely monotonic but also structurally nonlinear. At lower score ranges, the SHAP values are predominantly negative, whereas beyond an intermediate transition region the contribution becomes strongly positive. This pattern suggests that the model has learned a threshold-sensitive representation of credit quality, which is highly consistent with practical credit screening logic. The color distribution further indicates that the effect of CreditScore is modulated by unemployment status. For samples with similar credit scores, unemployed applicants tend to receive lower SHAP values than non-unemployed applicants, showing that the positive effect of credit quality is weakened when employment stability is absent.

The contribution of Employment Type_Unemployed remains strongly negative across samples, but its magnitude is visibly modulated by the credit score level, implying that the adverse effect of unemployment can be partially mitigated, though not eliminated, when credit quality is stronger.

**Figure 14:**
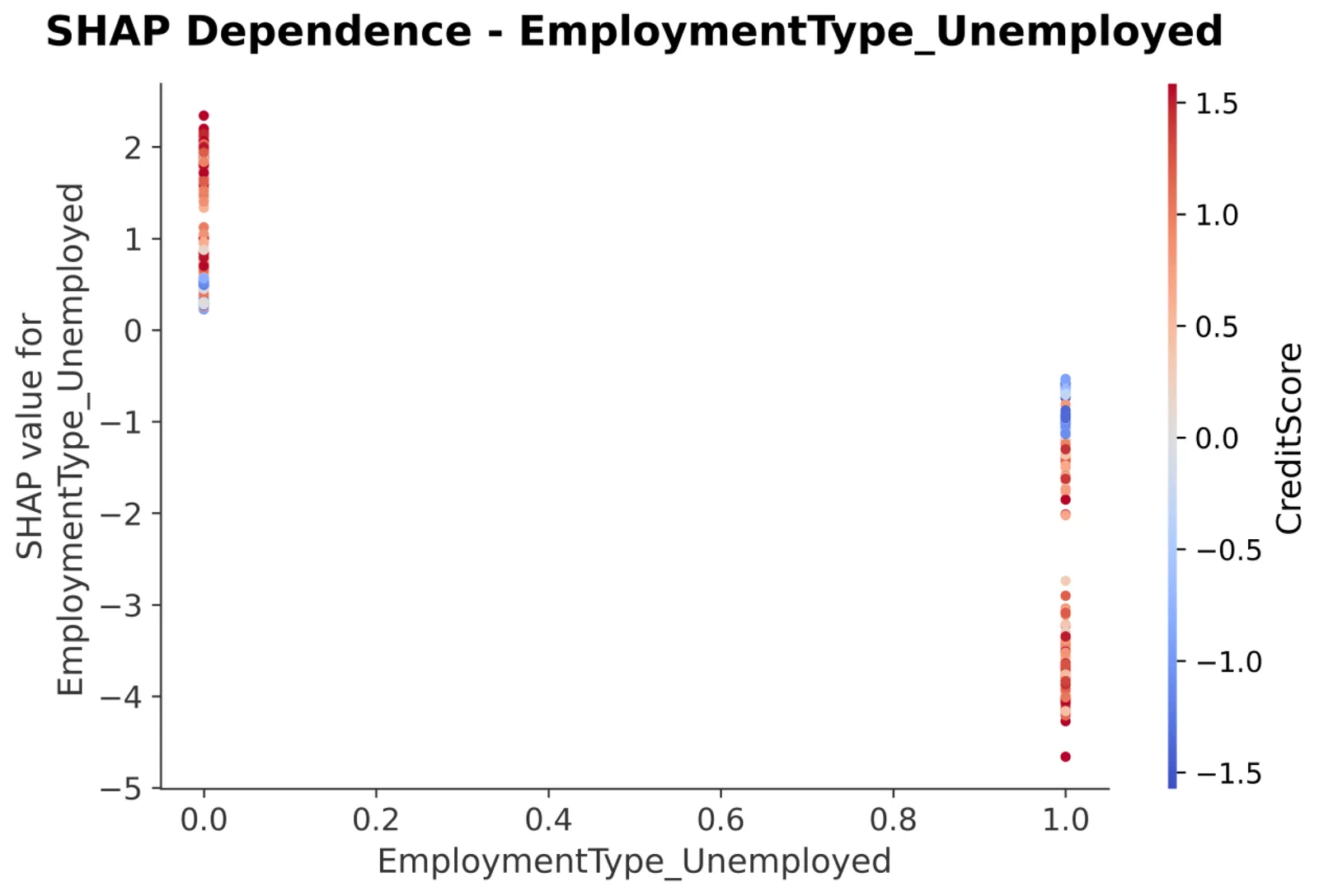
SHAP dependence plot of EmploymentType_Unemployed with interaction coloring by CreditScore.

Figure 14 confirms that unemployment status is one of the strongest negative signals in the model. When the EmploymentType_Unemployed indicator takes the active state, the SHAP value shifts markedly toward the negative range, demonstrating a stable suppressive effect on the approval prediction. The interaction coloring reveals that higher credit scores can reduce the magnitude of this negative contribution to some extent, but do not reverse its direction. This result indicates that the model does not treat unemployment as an isolated binary penalty; instead, it places unemployment within a broader conditional-risk context shaped by borrower credit quality.

A similar interaction pattern is observed for Income. Higher income values generally increase the predicted approval tendency, yet the extent of this positive contribution depends on the concurrent credit score.

**Figure 15:**
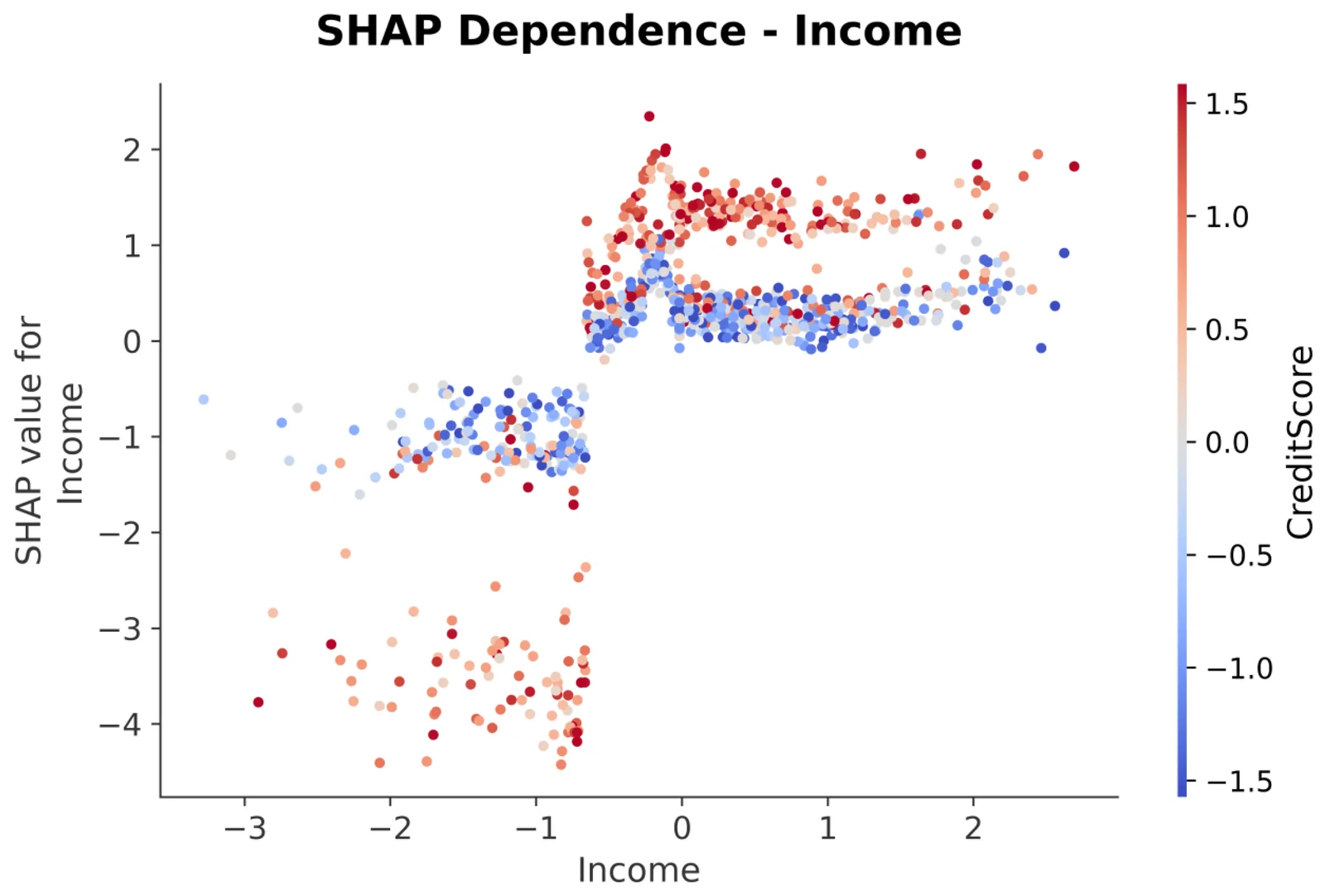
SHAP dependence plot of Income with interaction coloring by CreditScore.

Figure 15 shows that Income contributes to the model output in a generally positive and smoother manner than CreditScore. Lower income levels are associated with negative or near-zero SHAP values, whereas higher income levels increasingly shift the contribution toward the positive range. The interaction coloring indicates that this contribution is conditioned by credit score: applicants with stronger credit profiles tend to derive greater positive benefit from higher income, while lower credit quality weakens that effect. The result suggests that the model interprets income not as an isolated indicator of repayment capacity, but as a variable whose predictive meaning depends on the broader credit profile of the borrower.

These explanation results indicate that the proposed model has learned a conditional and interaction-aware risk mechanism rather than a purely additive decision rule, which strengthens the practical interpretability of the framework in credit assessment scenarios.

For a cross-model attribution comparison, SHAP TreeExplainer was applied to Random Forest and Gradient Boosting on the primary dataset, and their global feature rankings were compared with the transformer ranking on a fixed set of eight features.

**Figure 16:**
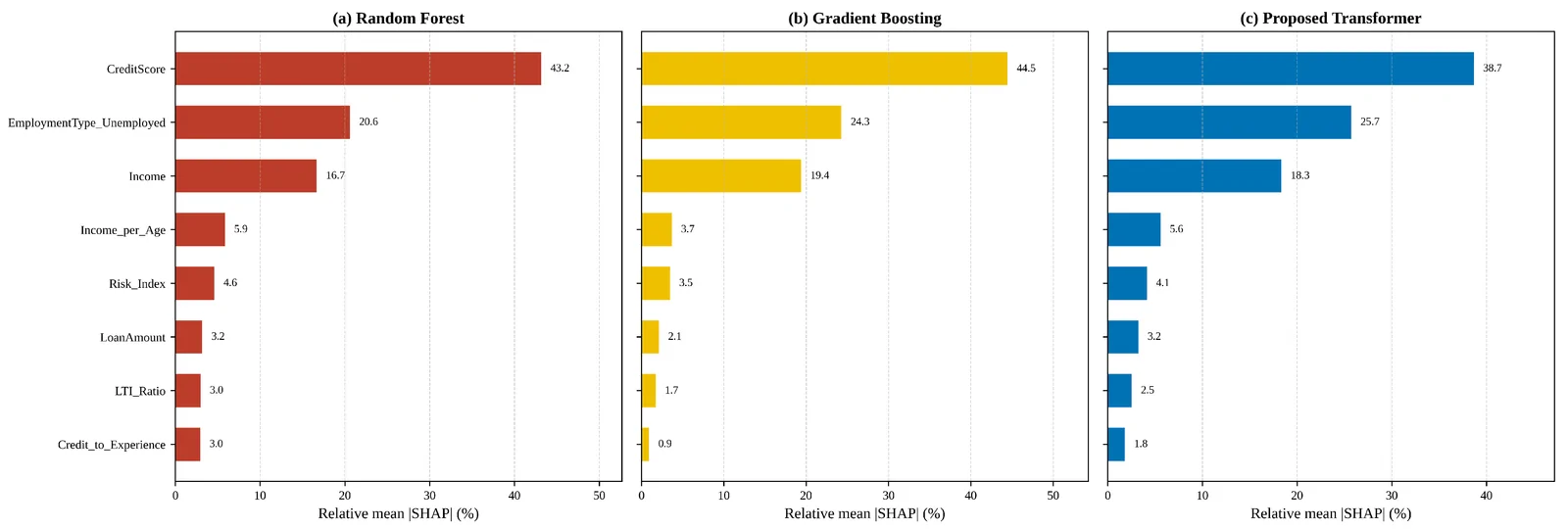
Comparison of SHAP-based global feature importance across Random Forest, Gradient Boosting, and the proposed transformer, expressed as each feature’s relative share of total mean |SHAP| within each model.

Table 11 and Figure 16 show that the same three features, CreditScore, EmploymentType_Unemployed, and Income, occupy the top three positions in the global SHAP ranking of all three classifiers, together accounting for roughly 80–90% of total attributed importance within each model. The ordering among the five remaining features is less consistent across models, and the raw mean |SHAP| magnitudes in Table 11 are not directly comparable across classifiers, since Random Forest, Gradient Boosting, and the transformer produce SHAP values on different output scales (probability, log-odds, and pre-sigmoid logit, respectively); the within-model percentage shares in Figure 16 provide the cross-model comparison. The identical top three predictors across all classifiers indicate that this attribution pattern primarily reflects the structure of the prediction task. As discussed in Section 5.2, SHAP provides a common model-agnostic explanation framework for both ensemble and transformer classifiers.

### 4.7. Supplementary Validation on a Real-Default Dataset

Table 12 summarizes the dataset validation coverage of this study before the supplementary validation is presented in detail.

These two datasets span a synthetic approval-decision proxy target and an authentic default-labeled dataset at roughly a six-fold difference in scale. Further validation across additional public and institutional credit datasets is required to establish broader generalizability.

The baseline comparison reported in Section 4.2 uses loan approval status as the prediction target on a dataset of 5000 records. Sensitivity of the comparative ranking to target definition and sample scale was evaluated on the UCI Default of Credit Card Clients dataset, whose binary target records whether a client defaults on payment in the following month. This dataset is substantially larger than the primary dataset. The three classifiers were retrained using the same imbalance-aware procedures as in the primary experiments. This supplementary validation covers the baseline performance comparison; the domain-informed ratio features, cross-attention and feature ablations, and SHAP analysis in Section 4.3, Section 4.4 and Section 4.6 remain specific to the primary dataset.

Table 13 shows that the supplementary dataset preserves a class imbalance ratio (approximately 78%/22%) similar in spirit to the primary dataset (77%/23%), while providing six times as many samples and, critically, a target variable that records an authentic financial outcome rather than a historical approval decision.

Table 14 shows that Random Forest and Gradient Boosting achieve broadly comparable performance on this dataset, each favoring a different point along the precision–recall trade-off: Random Forest attains the higher mean accuracy, precision, and F1-score, although its F1-score interval overlaps with that of Gradient Boosting, while Gradient Boosting attains the higher mean recall, ROC-AUC, and average precision, the latter two likewise overlapping with Random Forest within one standard deviation. Random Forest trains in under two seconds on average, and Gradient Boosting trains in a fraction of a second, whereas the proposed Cross-Attentional Tabular Transformer requires 7.38 s on average. The proposed transformer does not attain the best mean value on any of the six evaluated performance metrics: its accuracy, precision, and F1-score fall below Random Forest, its recall, ROC-AUC, and average precision fall below Gradient Boosting, and its training time exceeds both ensemble baselines by a wide margin. This pattern reproduces, on a dataset six times larger than the primary dataset and using an authentic repayment-default target rather than a historical approval decision, the qualitative finding already reported in Section 4.2: the two ensemble baselines remain preferable to the proposed transformer on both predictive performance and computational cost. Because the target variable and the dataset scale differ jointly between the primary and supplementary datasets, this validation cannot isolate which of the two factors is responsible for the outcome; it does, however, indicate that the comparative disadvantage of the proposed architecture observed in this study is not confined to the small, approval-labeled dataset used for the main experiments.

Several of the strongest predictors in this dataset, namely the six-month repayment status history (PAY_0 through PAY_6), directly encode recent repayment behavior rather than static demographic or financial attributes. This predictive structure differs materially from that of the primary dataset and plausibly contributes to the relatively higher recall obtained by Gradient Boosting. The supplementary validation reports the baseline comparison, while the ablation and SHAP analyses are reported for the primary dataset in Section 4.3, Section 4.4 and Section 4.6.

### 4.8. Sensitivity to Missingness and Class Skew

The comparisons reported above use the missingness pattern and class-skew ratio naturally present in the primary and supplementary datasets. Sensitivity of the low-cost baseline ranking was evaluated by retraining the six baselines from Section 4.2 (Random Forest, Gradient Boosting, XGBoost, LightGBM, CatBoost, and calibrated logistic regression) under two perturbed conditions. The increased-missingness condition raises the missingness rate of the affected fields beyond the levels in Table 2 while retaining the original 4000-sample, 23.03%-positive training set. The severe training-class-skew condition downsamples the training set to 3421 samples at a 10% positive rate, compared with approximately 23% in the original training set. This analysis covers the six low-cost baselines. Changes are calculated relative to each model’s own unperturbed single-fixed-split performance, a separate internal reference from the fixed-split and five-split results in Section 4.2 and Section 4.7.

Table 15 shows that all five tree-based and Gradient-Boosting methods degrade only modestly under increased missingness, with F1-score reductions of roughly 2–4 percentage points, and degrade by a comparable or smaller margin under severe training-class skew, several even showing a small positive ΔAUC and ΔAvg. Prec. under severe skew. Calibrated logistic regression is comparatively more sensitive to severe class skew, with an F1-score reduction of 0.1142, a substantially larger degradation than any of the tree-based or Gradient-Boosting methods, though its ROC-AUC is comparatively stable across both conditions. The near-zero or slightly positive ΔAUC observed for every model under severe training skew, despite the substantial drop in threshold-dependent metrics such as recall and F1-score, is consistent with the rank-based construction of ROC-AUC: because AUC evaluates the ordering of predicted scores across all thresholds rather than classification outcomes at a fixed cut-off, it is comparatively insensitive to the class prior used during training, whereas accuracy, precision, recall, and F1-score are evaluated at a fixed threshold and therefore shift more directly with the training class balance. This divergence between threshold-independent and threshold-dependent metrics under severe skew is expected rather than anomalous, and calibrated logistic regression illustrates it most clearly, with a large F1-score reduction alongside a negligible change in AUC. This pattern indicates that the tree-based and Gradient-Boosting baselines used throughout this study are reasonably robust to the specific missingness and skew conditions tested here, while a simple linear baseline is comparatively more fragile in its threshold-dependent metrics. This analysis does not include the proposed transformer and therefore does not establish its robustness under the same perturbations, which remains untested and is noted as a limitation in Section 5.2.

## 5. Discussion and Conclusions

### 5.1. Main Findings

This study developed a Cross-Attentional Tabular Transformer framework for credit risk assessment and examined its performance on structured loan application data under an imbalanced classification setting. The experimental results showed that Gradient Boosting achieved the best overall fixed-threshold classification performance, while Random Forest obtained the highest ROC-AUC. The proposed transformer-based model remained competitive, confirming the feasibility of attention-based tabular learning for heterogeneous credit data, although it did not exceed the strongest tree-based baselines under the present experimental conditions.

The analysis further indicated that the predictive structure of the task was dominated by a limited number of financially meaningful variables, particularly CreditScore, EmploymentType_Unemployed, and Income. The explainability results showed that the proposed framework learned interpretable and interaction-aware decision patterns rather than relying on diffuse or weak predictors. Two ablation studies further confirmed the contribution of the individual framework components. The cross-attention ablation showed that the bidirectional cross-attention design improves F1-score from 0.8865 to 0.9043, ROC-AUC from 0.9274 to 0.9386, and reduces false negatives from 35 to 28 compared with a vanilla TabTransformer baseline. The feature ablation showed that removing the three engineered ratio features reduces F1-score from 0.9043 to 0.8840, ROC-AUC from 0.9386 to 0.9251, and increases false negatives from 28 to 36. These findings establish that the overall performance of the proposed framework reflects the combined contribution of the cross-attentional representation mechanism and the domain-informed financial feature construction, while ensemble methods remain highly competitive for moderate-scale static tabular credit prediction.

The conclusions derive from experiments on a structured benchmark dataset of 5000 loan application records with a specific demographic and geographic scope. The results characterize the relative properties and trade-offs of the compared approaches under these conditions. Because loan approval status is the prediction target, the primary models evaluate historical lending decisions rather than actual repayment outcomes. Broader applicability across larger data scales and different borrower populations requires additional empirical investigation. In the supplementary validation on a larger dataset with an authentic default target, both ensemble baselines again outperform the transformer and train substantially faster, although the relative standing of Random Forest and Gradient Boosting differs between the datasets. The target definition and scale change jointly in the supplementary dataset, so their individual effects cannot be isolated.

### 5.2. Discussion and Future Work

The central result of this study is that, for the moderate-scale, static, single-target credit dataset examined here, the two ensemble baselines outperform the proposed Cross-Attentional Tabular Transformer on every evaluated metric while requiring more than two orders of magnitude less training time. Under the five-split robustness protocol, Random Forest, Gradient Boosting, XGBoost, LightGBM, and CatBoost also exceed the transformer’s single-split performance, whereas calibrated logistic regression does not. The evaluated scope covers these classical and contemporary tree ensembles and the linear baseline; deep tabular architectures such as TabNet, FT-Transformer, SAINT, and TabPFN-style methods remain outside the comparison. Within this scope, the methodological contribution is the cross-attentional design principle and its empirical characterization.

The bidirectional cross-attention mechanism provides an explicit, inspectable structure for exchanges between numerical and categorical feature groups, and the ablation study shows measurable improvement over a vanilla concatenation-based self-attention baseline. Across all three classifiers, the SHAP analysis in Table 11 and Figure 16 identifies the same three dominant features, indicating a stable attribution pattern across architectures. Future evaluation can examine the cross-attention design with incomplete data, sequential borrower histories, and pretraining on unlabeled records.

Several limitations bound these findings. The primary dataset comprises 5000 records with a specific demographic and geographic scope, below the scale at which transformer architectures typically show their full representational advantage. The tree-based and Gradient-Boosting baselines are reasonably robust to increased missingness and severe training-class skew, while transformer performance under the same perturbations remains unevaluated. The prediction target reflects historical lending decisions rather than realized repayment outcomes. A supplementary validation with an authentic default target again finds both ensembles ahead of the transformer, although that dataset also differs in scale and feature composition. The findings therefore describe methodological results under the evaluated conditions rather than transferable risk rules for other institutional or geographic settings.

The relationship between sample size and algorithm suitability remains open in the broader literature. Hollmann et al. [4] identify conditions under which transformer-based tabular models are competitive with tree ensembles. The primary and supplementary results here do not show the performance gap closing, but the two evaluated scales do not resolve the general scaling relationship. The training-time comparison reflects a single run on a small static dataset. High-dimensional or sequential inputs and transfer learning from unlabeled records remain outside the evaluated setting.

Future work can extend the comparison to richer credit scenarios involving temporal borrower behavior and multi-source information integration. The current evidence covers variation in dataset scale and target-variable authenticity, with both ensemble baselines ahead of the proposed transformer in the two evaluated datasets.

## Figures and Tables

**Table 1:** Positioning against representative studies in credit risk prediction (qualitative comparison).

Study (Ref.)	Imbalance Handling	Ensemble/Hybrid Modeling	Explainability/Interpretability	Cost-Sensitive/ Risk-Aware Design	Decision-Oriented Evaluation	Practical Financial Relevance
Review studies [9,13]	partial	partial	partial		partial	✓
Ensemble-based studies [5,7,15,16]	partial	✓	partial		partial	✓
Sampling-oriented studies [12,14]	✓	partial			partial	✓
Explainable credit risk studies [2,3,10]	partial	partial	✓		partial	✓
Specialized/advanced modeling studies [4,11,17]	partial	partial	partial	partial	partial	partial
**This work**	**✓ (integrated resampling)**	**✓ (systematic ML/ensemble comparison)**	**✓ (interpretable model analysis)**	**✓ (cost-sensitive learning)**	**✓ (F1, recall, ROC–AUC)**	**✓ (financially aligned** **framework)**

Checkmarks in the “This work” row identify the design elements incorporated into the proposed framework.

**Table 2:** Summary of the raw credit risk dataset.

Variable	Type	Description	Missing
Age	Numerical	Age of the applicant	0
Income	Numerical	Applicant income	196
LoanAmount	Numerical	Requested loan amount	0
CreditScore	Numerical	Credit score of the applicant	194
YearsExperience	Numerical	Years of work experience	0
Gender	Categorical	Gender of the applicant	0
Education	Categorical	Education level	198
City	Categorical	City of residence	0
EmploymentType	Categorical	Employment status/type	0
LoanApproved	Binary target	Credit approval outcome	0

**Table 3:** Descriptive statistics of numerical variables in the raw dataset.

Variable	Mean	Std	Min	25%	Median	75%	Max
Age	43.48	14.43	18	31	44	56	69
Income	50,530.81	14,929.49	−5000.00	40,041.50	50,627.50	60,416.00	99,872.00
LoanAmount	20,087.29	8543.40	−10,000.00	12,853.25	20,071.50	27,376.25	48,000.00
CreditScore	575.62	144.61	300	449	577	699	849
YearsExperience	19.52	11.50	0	10	20	29	39

**Table 4:** Class distribution of the raw, training, and testing datasets.

Dataset	Not Approved	Approved	Not Approved (%)	Approved (%)	Total
Raw dataset	3849	1151	76.98	23.02	5000
Training set	3079	921	76.98	23.02	4000
Test set	770	230	77.00	23.00	1000

**Table 5:** Structure of the processed data used for traditional machine learning models.

Feature Group	Variables/Encoded Fields	Dim.
Standardized numerical	Age, Income, LoanAmount, CreditScore, YearsExperience	5
Engineered continuous	LTI_Ratio, Credit_to_Experience, Risk_Index, Income_per_Age	4
Gender encoding	Gender_Female, Gender_Male	2
Education encoding	Education_Bachelors, Education_High School, Education_Masters, Education_PhD	4
City encoding	City_Chicago, City_Houston, City_New York, City_San Francisco	4
EmploymentType encoding	EmploymentType_Salaried, EmploymentType_Self-Employed, EmploymentType_Unemployed	3
Pre-engineering total	Original numerical + encoded categorical variables	18
Final predictive total	Original numerical + engineered continuous + encoded categorical variables	22
Target variable	LoanApproved	1

**Table 6:** Robustness of low-cost tabular baselines on the primary approval-decision dataset (mean ± standard deviation across five stratified 80/20 splits).

Model	Acc.	Prec.	Recall	F1	AUC	Avg. Prec.	Training Time (s)
Random Forest	0.9674 ± 0.0036	0.9576 ± 0.0112	0.8983 ± 0.0198	0.9268 ± 0.0088	0.9449 ± 0.0092	0.9066 ± 0.0114	0.4759 ± 0.0209
Gradient Boosting	0.9672 ± 0.0041	0.9558 ± 0.0087	0.8991 ± 0.0200	0.9264 ± 0.0098	0.9430 ± 0.0151	0.9056 ± 0.0105	0.5868 ± 0.0026
XGBoost	0.9674 ± 0.0044	0.9575 ± 0.0079	0.8983 ± 0.0223	0.9268 ± 0.0106	0.9422 ± 0.0151	0.9095 ± 0.0068	0.6928 ± 0.0880
LightGBM	0.9668 ± 0.0040	0.9540 ± 0.0084	0.8991 ± 0.0198	0.9256 ± 0.0095	0.9437 ± 0.0129	0.9119 ± 0.0088	0.0473 ± 0.0016
CatBoost	0.9622 ± 0.0056	0.9340 ± 0.0057	0.8991 ± 0.0216	0.9162 ± 0.0131	0.9437 ± 0.0105	0.9011 ± 0.0124	0.0803 ± 0.0097
Calibrated Logistic Regression	0.8828 ± 0.0047	0.7813 ± 0.0177	0.6826 ± 0.0432	0.7277 ± 0.0191	0.9174 ± 0.0083	0.8130 ± 0.0118	0.0385 ± 0.0284

These six baselines were retrained and re-evaluated across five independent stratified 80/20 splits of the primary dataset, the same splitting protocol used for the supplementary validation in Section 4.7. The transformer result reflects the single fixed split used in the primary comparison. Cross-protocol comparisons between that estimate and the five-split baseline means are descriptive; transformer split-to-split stability remains a topic for future evaluation.

**Table 7:** Ablation study results for the bidirectional cross-attention mechanism.

Model Variant	Attention Mechanism	Acc.	Prec.	Recall	F1	AUC	Avg. Prec.
Vanilla TabTransformer	Concatenated tokens + self-attention	0.9450	0.9126	0.8618	0.8865	0.9274	0.8956
Proposed Transformer	Bidirectional cross-attention	0.9530	0.9212	0.8880	0.9043	0.9386	0.9128
Improvement (Δ)	—	+0.0080	+0.0086	+0.0262	+0.0178	+0.0112	+0.0172

**Table 8:** Confusion matrices for the vanilla TabTransformer and the proposed cross-attention model on the test set.

Model Variant	True Class	Predicted: Not Approved (0)	Predicted: Approved (1)
Vanilla TabTransformer	Not Approved (0)	729	21
	Approved (1)	35	215
Proposed Transformer	Not Approved (0)	731	19
	Approved (1)	28	222

**Table 9:** Ablation study results for the engineered financial ratio features.

Model Setting	Engineered Features	Acc.	Prec.	Recall	F1	AUC	Avg. Prec.
Proposed Transformer w/o ratio features	Removed	0.9440	0.9138	0.8560	0.8840	0.9251	0.8914
Proposed Transformer with ratio features	Included	0.9530	0.9212	0.8880	0.9043	0.9386	0.9128
Improvement (Δ)	—	+0.0090	+0.0074	+0.0320	+0.0203	+0.0135	+0.0214

**Table 10:** Confusion matrices for the proposed model with and without engineered financial ratio features on the test set.

Model Setting	True Class	Predicted: Not Approved (0)	Predicted: Approved (1)
Proposed Transformer	Not Approved (0)	730	20
*w*/*o* ratio features	Approved (1)	36	214
Proposed Transformer	Not Approved (0)	731	19
with ratio features	Approved (1)	28	222

**Table 11:** SHAP-based global importance comparison for the fixed eight features displayed in Figure 16.

Feature (Fixed Order, as in Figure 16)	Random Forest	Gradient Boosting	Proposed Transformer
CreditScore	0.217	1.831	2.150
EmploymentType_Unemployed	0.104	1.000	1.430
Income	0.084	0.798	1.020
Income_per_Age	0.029	0.152	0.310
Risk_Index	0.023	0.142	0.230
LoanAmount	0.016	0.087	0.180
LTI_Ratio	0.015	0.071	0.140
Credit_to_Experience	0.015	0.038	≈0.10 ^†^

Values denote mean absolute SHAP values and are rounded to three decimal places; the percentage shares in Figure 16 were calculated from the corresponding full-precision values before rounding. The eight features are fixed to match those displayed in Figure 16, and the row order reflects the proposed transformer’s descending ranking. The baseline columns retain this fixed order, so rows 4–8 do not necessarily follow each baseline model’s individual ranking; the top three rows coincide across all models. Random Forest and Gradient Boosting values were computed using SHAP TreeExplainer on the primary 5000-sample dataset, and the proposed-transformer values correspond to the analysis in Figure 12. ^†^ The Credit_to_Experience value in the proposed-transformer column is an approximation inferred from its ranking position in Figure 12.

**Table 12:** Completed dataset validation coverage in this study.

Dataset	Task Label	Sample Size	Validation Role
Primary LoanApproved dataset	Historical loan approval decision	5000	Primary approval-decision modeling task
UCI Default of Credit Card Clients	Default payment next month	30,000	Supplementary real-default validation

**Table 13:** Description of the supplementary real-default validation dataset.

Item	Description		
Data source	UCI Default of Credit Card Clients dataset		
Prediction target	default_payment_next_month		
Target meaning	Whether a credit card client defaults on payment in the following month		
Task type	Binary credit default prediction		
Sample size	30,000 records		
Train/test protocol	Stratified 80%/20% split, repeated across five independent random seeds (11, 22, 33, 42, 55)		
Training set size	24,000 records per split		
Test set size	6000 records per split		
Number of features	23 original features		
ID column	Removed before modeling		
Missing values	None observed in the cleaned dataset		
Positive class	Default = 1		
Negative class	Non-default = 0		
**Class distribution (representative split, seed 42; stratification yields essentially the same proportions across all five seeds):**			
**Dataset Split**	**Non-Default, n (%)**	**Default, n (%)**	**Total**
Raw dataset	23,364 (77.88%)	6636 (22.12%)	30,000
Training set	18,691 (77.88%)	5309 (22.12%)	24,000
Test set	4673 (77.88%)	1327 (22.12%)	6000

**Table 14:** Main performance comparison on the supplementary real-default dataset (mean ± standard deviation across five stratified 80/20 splits, seeds 11, 22, 33, 42, 55; bold indicates the best mean value per metric).

Model	Acc.	Prec.	Recall	F1	AUC	Avg. Prec.	Training Time (s)
Random Forest	**0.8042 ± 0.0037**	**0.5609 ± 0.0096**	0.5286 ± 0.0061	**0.5442 ± 0.0069**	0.7753 ± 0.0074	0.5490 ± 0.0134	1.7833 ± 0.0717
Gradient Boosting	0.7615 ± 0.0055	0.4712 ± 0.0086	**0.6368 ± 0.0105**	0.5415 ± 0.0062	**0.7797 ± 0.0054**	**0.5494 ± 0.0120**	**0.2490 ± 0.0130**
Proposed Transformer	0.7809 ± 0.0101	0.5051 ± 0.0210	0.5604 ± 0.0158	0.5309 ± 0.0087	0.7697 ± 0.0075	0.5358 ± 0.0135	7.3840 ± 1.2211

**Table 15:** Sensitivity of low-cost tabular baselines to increased missingness and severe training-class skew on the primary dataset, reported as perturbed performance and change (Δ) relative to each model’s own unperturbed baseline.

Condition	Model	Acc.	ΔAcc.	F1	ΔF1	AUC	ΔAUC	Avg.P.	ΔAvg.P.
Incr. missingness	Random Forest	0.9530	−0.0100	0.8924	−0.0245	0.9321	−0.0058	0.8682	−0.0331
	Gradient Boosting	0.9470	−0.0160	0.8814	−0.0355	0.9237	−0.0019	0.8652	−0.0276
	XGBoost	0.9490	−0.0140	0.8859	−0.0306	0.9267	−0.0001	0.8788	−0.0194
	LightGBM	0.9490	−0.0130	0.8859	−0.0289	0.9213	−0.0061	0.8762	−0.0206
	CatBoost	0.9490	−0.0090	0.8854	−0.0213	0.9313	−0.0044	0.8837	−0.0038
	Calibrated LR	0.8790	−0.0070	0.7206	−0.0179	0.9075	−0.0033	0.8017	−0.0091
Severe training skew	Random Forest	0.9590	−0.0040	0.9062	−0.0107	0.9415	+0.0036	0.8922	−0.0091
	Gradient Boosting	0.9600	−0.0030	0.9103	−0.0066	0.9370	+0.0114	0.9022	+0.0094
	XGBoost	0.9610	−0.0020	0.9124	−0.0041	0.9382	+0.0114	0.9067	+0.0085
	LightGBM	0.9610	−0.0010	0.9120	−0.0028	0.9374	+0.0100	0.9063	+0.0095
	CatBoost	0.9550	−0.0030	0.9007	−0.0060	0.9398	+0.0041	0.8955	+0.0080
	Calibrated LR	0.8640	−0.0220	0.6243	−0.1142	0.9114	+0.0006	0.8103	−0.0005

“Calibrated LR” denotes calibrated logistic regression; “Avg.P.” denotes average precision. Each model’s unperturbed baseline provides the internal reference for Δ; positive values indicate improvement under the perturbation and negative values indicate degradation. The table reports a separate sensitivity analysis for the low-cost tabular baselines, and each row reflects a single perturbed training run rather than a five-split average.

## Data Availability

The data presented in this study are available on request from the corresponding author due to privacy requirements.

## Abbreviations

The following abbreviations are used in this manuscript:

AUC	Area Under the Curve
BCE	Binary Cross-Entropy
FFN	Feed-Forward Network
LTI	Loan-to-Income
MHA	Multi-Head Attention
MI	Mutual Information
PCA	Principal Component Analysis
PR	Precision–Recall
RF	Random Forest
ROC	Receiver Operating Characteristic
SHAP	SHapley Additive exPlanations
WBCE	Weighted Binary Cross-Entropy

## Appendix A. Additional Experimental Details

### Appendix A.1. Ancillary Feature-Screening Experiment

Table A1 summarizes an ancillary feature-screening experiment based on a reduced candidate set and an independently configured mutual-information and Random Forest analysis. Figure 4 presents the normalized ranking from the main feature-analysis pipeline; the two analyses use different candidate sets and configurations and are interpreted separately. The final model specification follows the four engineered-feature definitions in Section 3.3.1: LTI_Ratio, Credit_to_Experience, Risk_Index, and Income_per_Age. Credit_to_Loan is confined to the alternative candidate set in the ancillary experiment. MI is estimated on the ancillary training subset using a non-parametric *k*-nearest-neighbor estimator, with the five original numerical features providing within-experiment reference values.

**Table A1:** Candidate-feature relevance results from a separate ancillary screening experiment.

Feature	Definition	MI (nats)	RF Importance
Original numerical features			
CreditScore	Raw credit score of the applicant	0.184	0.321
Income	Gross annual income	0.089	0.158
LoanAmount	Requested loan principal	0.024	0.047
Age	Age of the applicant	0.018	0.031
YearsExperience	Accumulated years of employment	0.015	0.028
Engineered interaction features			
LTI_Ratio	$L_{i}/\left(I_{i}+\varepsilon \right)$: loan-to-income burden	0.043	0.079
Credit_to_Exp	$C_{i}/\left(E_{i}+\varepsilon \right)$: credit quality per experience year	0.067	0.112
Credit_to_Loan	$C_{i}/\left(L_{i}+\varepsilon \right)$: ancillary alternative candidate	0.038	0.064

### Appendix A.2. Model Hyperparameter Configurations

Table A2 lists the key hyperparameters for the three models evaluated in this study. The two ensemble baselines use their scikit-learn default configurations with class-weighting enabled to address label imbalance. The proposed transformer applies focal–loss training with class-dependent balancing.

**Table A2:** Hyperparameter configurations of the three compared models.

Setting	Value
Random Forest	
Number of trees	100
Maximum depth	Unlimited
Minimum samples per split	2
Feature subset per split	$\sqrt{d}$
Class weighting	Balanced (inverse frequency)
Gradient Boosting	
Number of estimators	100
Learning rate	0.1
Maximum tree depth	3
Subsample ratio	1.0
Loss	Log-loss (binary deviance)
Cross-Attentional Tabular Transformer	
Token embedding dimension (*d*)	32
Cross-attention heads	4
Encoder layers	2
FFN hidden dimension	64
Dropout rate	0.1
Training loss	Focal loss (γ=2, class-weighted)
Optimizer	Adam
Learning rate	$1\times 10^{-3}$
Batch size	64
Training epochs	50

### Appendix A.3. Per-Class Classification Performance on the Test Set

Table A3 reproduces the per-class values underlying Figure 7 in exact numeric form, for reference and reproducibility; the corresponding interpretation is given in Section 4.2 and is not repeated here.

**Table A3:** Per-class precision, recall, and F1-score on the 1000-sample test set (770 Not Approved, 230 Approved).

Model	Class	Precision	Recall	F1-Score
Random Forest	Not Approved (0)	0.967	0.986	0.976
	Approved (1)	0.948	0.887	0.916
Gradient Boosting	Not Approved (0)	0.967	0.987	0.977
	Approved (1)	0.953	0.887	0.919
Transformer (proposed)	Not Approved (0)	0.962	0.978	0.970
	Approved (1)	0.922	0.870	0.895
